# A variant *ECE1* allele contributes to reduced pathogenicity of *Candida albicans* during vulvovaginal candidiasis

**DOI:** 10.1371/journal.ppat.1009884

**Published:** 2021-09-10

**Authors:** Junyan Liu, Hubertine M. E. Willems, Emily A. Sansevere, Stefanie Allert, Katherine S. Barker, David J. Lowes, Andrew C. Dixson, Zhenbo Xu, Jian Miao, Christian DeJarnette, Helene Tournu, Glen E. Palmer, Jonathan P. Richardson, Francisco N. Barrera, Bernhard Hube, Julian R. Naglik, Brian M. Peters

**Affiliations:** 1 School of Food Science and Engineering, Guangdong Province Key Laboratory for Green Processing of Natural Products and Product Safety, South China University of Technology, Guangzhou, China; 2 Department of Clinical Pharmacy and Translational Science, College of Pharmacy, University of Tennessee Health Science Center, Memphis, Tennessee, United States of America; 3 Department of Microbial Pathogenicity Mechanisms, Hans Knöll Institute, Leibniz Institute for Natural Product Research and Infection Biology, Jena, Germany; 4 Department of Biochemistry & Cellular and Molecular Biology, University of Tennessee, Knoxville, Tennessee, United States of America; 5 Graduate Program in Pharmaceutical Sciences, College of Graduate Health Sciences, University of Tennessee Health Science Center, Memphis, Tennessee, United States of America; 6 Integrated Program in Biomedical Sciences, College of Graduate Health Sciences, University of Tennessee Health Science Center, Memphis, Tennessee, United States of America; 7 Department of Microbiology, Immunology, and Biochemistry, College of Medicine, University of Tennessee Health Science Center, Memphis, Tennessee, United States of America; 8 Centre for Host-Microbiome Interactions, Faculty of Dentistry, Oral & Craniofacial Sciences, King’s College London, London, United Kingdom; 9 Institute of Microbiology, Friedrich-Schiller-University Jena, Jena, Germany; University of Maine, UNITED STATES

## Abstract

Vulvovaginal candidiasis (VVC), caused primarily by the human fungal pathogen *Candida albicans*, results in significant quality-of-life issues for women worldwide. Candidalysin, a toxin derived from a polypeptide (Ece1p) encoded by the *ECE1* gene, plays a crucial role in driving immunopathology at the vaginal mucosa. This study aimed to determine if expression and/or processing of Ece1p differs across *C*. *albicans* isolates and whether this partly underlies differential pathogenicity observed clinically. Using a targeted sequencing approach, we determined that isolate 529L harbors a similarly expressed, yet distinct Ece1p isoform variant that encodes for a predicted functional candidalysin; this isoform was conserved amongst a collection of clinical isolates. Expression of the *ECE1* open reading frame (ORF) from 529L in an SC5314-derived *ece1*Δ/Δ strain resulted in significantly reduced vaginopathogenicity as compared to an isogenic control expressing a wild-type (WT) *ECE1* allele. However, *in vitro* challenge of vaginal epithelial cells with synthetic candidalysin demonstrated similar toxigenic activity amongst SC5314 and 529L isoforms. Creation of an isogenic panel of chimeric strains harboring swapped Ece1p peptides or HiBiT tags revealed reduced secretion with the ORF from 529L that was associated with reduced virulence. A genetic survey of 78 clinical isolates demonstrated a conserved pattern between Ece1p P2 and P3 sequences, suggesting that substrate specificity around Kex2p-mediated KR cleavage sites involved in protein processing may contribute to differential pathogenicity amongst clinical isolates. Therefore, we present a new mechanism for attenuation of *C*. *albicans* virulence at the *ECE1* locus.

## Author summary

Vulvovaginal candidiasis (VVC), caused primarily by the human fungal pathogen *Candida albicans*, results in significant quality-of-life issues for women worldwide. Candidalysin, a toxin derived from a polypeptide (Ece1p) encoded by the *ECE1* gene, plays a crucial role in driving disease pathology. In this study, we aimed to determine if genetic variation of *ECE1* existed across a collection of *C*. *albicans* clinical isolates. Using a targeted sequencing approach, we identified a conserved variant candidalysin isoform. We then constructed isogenic chimeric strains harboring swapped Ece1p peptides or HiBiT tags between representative isoforms and showed impaired secretion with the variant that was associated with reduced virulence in vitro and in vivo. We also elucidated a conserved pattern between key sequences near lysine-arginine cleavage sites involved in Ece1p polypeptide processing by the Kex2p enzyme that correlate with predicted and experimental candidalysin secretion. Therefore, we present a novel mechanism for attenuation of *C*. *albicans* virulence at the *ECE1* locus that may partly underlie differential clinical outcomes and contribute to the balance between commensal and pathogen at the vaginal mucosa.

## Introduction

Vulvovaginal candidiasis (VVC), caused primarily by the human fungal pathogen *Candida albicans*, results in significant quality-of-life issues for women worldwide [1]. In fact, it has been estimated that 75% of all women will experience at least one episode of VVC in their lifetime [2]. VVC has recently been described as an immunopathology, in which robust innate host defense mechanisms (e.g. neutrophil migration and pro-inflammatory cytokines) drive disease symptoms, including itching, burning, and soreness of the vaginal mucosa [3]. The host molecular mechanisms partially underpinning these outcomes have recently been linked to activation of the NLRP3 inflammasome, a damage-sensing immune complex that ultimately leads to secretion of bioactive interleukin-1 beta (IL-1β) [4,5,6]. While the intersection of host genetics, hormonal signaling, and lifestyle influence VVC susceptibility or relapse, isolate-specific fungal factors likely also play a significant role in disease progression and severity [7,8].

Using a murine model of VVC, our labs have collectively made several recent observations regarding *C*. *albicans* vaginopathogenicity [4,9,10,11]. As a polymorphic fungus, the capacity to switch from yeast to hyphal forms is regarded as the primary virulence attribute of *C*. *albicans* [12]. Using hypha-deficient mutant strains, we demonstrated that the yeast-to-hypha switch is crucial for eliciting mucosal damage and concomitant neutrophil recruitment and pro-inflammatory signaling at the vaginal interface [10]. These results largely recapitulated observations made using similar hypha-deficient mutants in the context of *in vitro* infection of vaginal epithelial cells. However, it remained unclear as to whether altered morphology alone was sufficient to drive these responses. A seminal paper by Moyes, et al. began to disentangle this important question by identifying candidalysin, a hypha-specific peptide toxin capable of inducing damage, MAPK signaling, and cytokine/chemokine secretion at mucosal surfaces [13]. Similar to a model of oropharyngeal candidiasis (OPC), expression of candidalysin is critical for robust induction of immunopathology during murine VVC, as strains lacking candidalysin failed to induce characteristic neutrophil recruitment and release of mucosal damage biomarkers despite colonizing the vaginal mucosa and forming hyphae *in vivo* [11]. Moreover, candidalysin is both sufficient and necessary to activate the NLRP3 inflammasome [14,15,16]. Thus, transition from yeast-to-hypha and subsequent candidalysin secretion are key fungal factors driving VVC immunopathology.

Candidalysin is encoded by the *ECE1* gene as a 271 amino acid (AA) polypeptide. Analysis of the protein sequence reveals interspersing of 7 arginine-lysine (KR) repeats throughout, which serve as cleavage substrates for the *C*. *albicans* Kexin-like protease Kex2p [13,17]. Thus, as Ece1p moves through the endoplasmic reticulum and Golgi apparatus, Kex2p generates 8 individual peptides that are released into the extracellular environment. Of these, peptide 3 was coined candidalysin based on its toxin-like properties. Alanine scanning revealed that efficient cleavage of KR residues immediately flanking the candidalysin sequence (i.e. peptide 2 (position R61) and peptide 4 (position R93)) are necessary for toxin activity on oral epithelial cells and for full virulence in a mouse model of OPC [18]. These studies underscore the crucial role of efficient Ece1p processing for optimal pathogenicity.

While significant attention has been traditionally given to identifying host factors that may contribute to VVC susceptibility, understanding how genetic differences in *C*. *albicans* clinical isolates may alter candidalysin expression, processing, and vaginopathogenicity is not well understood. A study by Rahman, et al. identified that the clinical isolate 529L (originally isolated from a case of oral candidiasis) exhibited a hyper-colonization phenotype in a model of low-dose estrogen concurrent oral and vaginal candidiasis [19]. Moreover, despite observing hyphal growth *in vivo*, there was comparatively little histological signs of disease during 529L colonization, which differed dramatically from challenge with the widely used reference isolate SC5314 [20].

Therefore, using strains SC5314 and 529L, the objective of this study was to determine whether expression and/or processing of candidalysin differs across *C*. *albicans* isolates which may partly explain differential pathogenicity. Using a combination of genetic, immunologic, and biochemical approaches, we demonstrate that strain 529L harbors a similarly expressed, yet distinct Ece1p isoform variant that encodes for a functional candidalysin. Use of synthetic peptides demonstrate similar toxigenic activity of the variant as to that found in SC5314. However, expression of the entire *ECE1* open reading frame (ORF) from 529L in an SC5314-based *ece1*Δ/Δ background results in significantly reduced vaginopathogenicity as compared to an isogenic control transformed with the SC5314 *ECE1* ORF. Creation of an isogenic panel of strains with swapped Ece1p peptides reveal a crucial role for Kex2p substrate specificity in driving vaginopathogenicity. Therefore, we present a novel mechanism contributing to attenuation of *C*. *albicans* virulence at the *ECE1* locus.

## Results

### *C*. *albicans* strain 529L elicits reduced vaginal immunopathology

**Fig 1:**
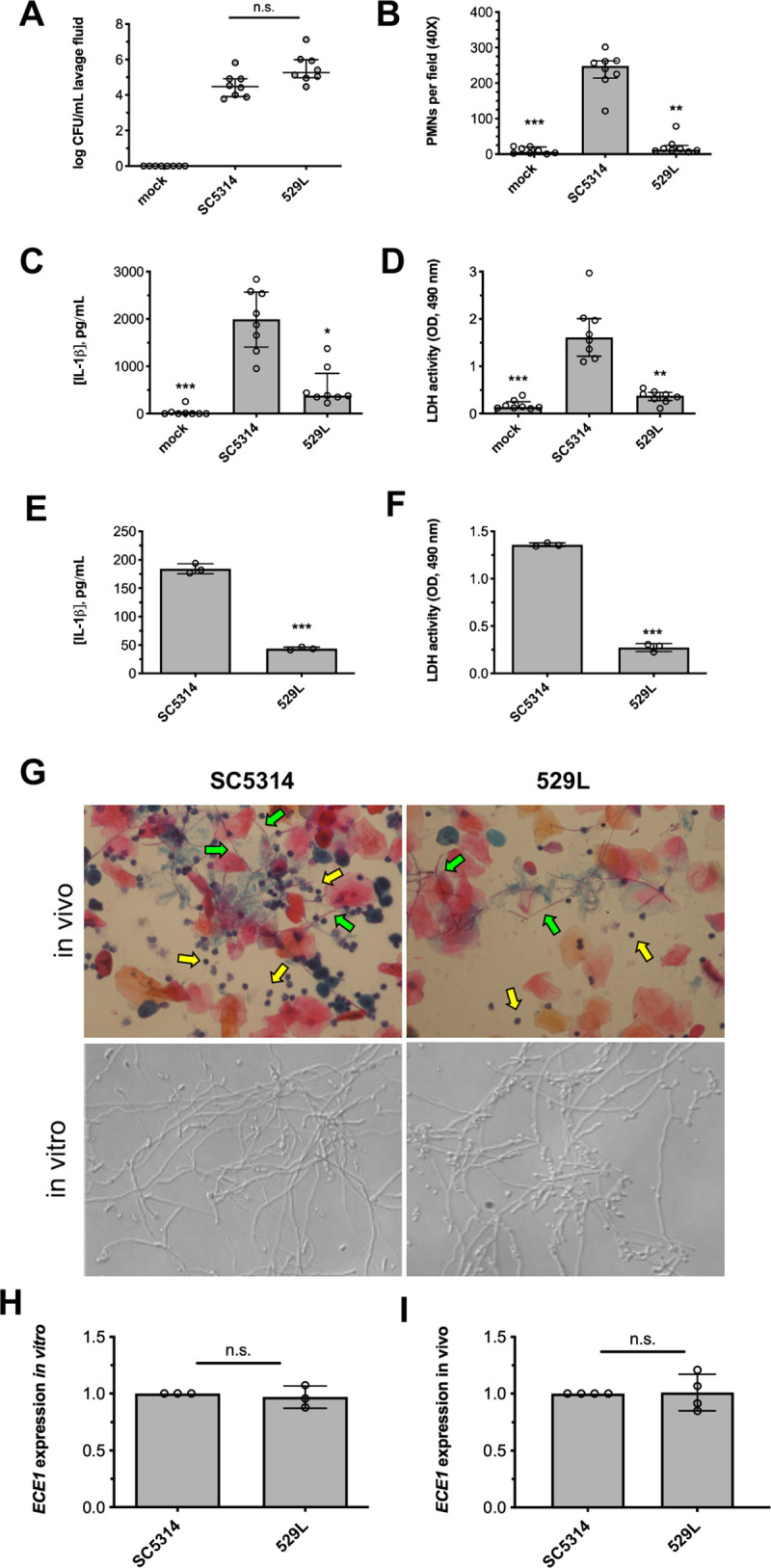
*C*. *albicans* isolate 529L elicits less vaginal immunopathology. WT strain SC5314 and clinical isolate 529L were intravaginally inoculated into estrogen-treated C57BL/6 mice. Mice (n = 8 per group) underwent vaginal lavage at d 3 post-infection and lavage fluids assessed for **(A)** CFU by microbiological plating (median ± interquartile range), **(B)** PMN recruitment by microscopy (median ± interquartile range), **(C)** IL-1β by ELISA (median ± IQR), and **(D)** tissue damage by LDH assay (median ± IQR). Statistical significance was assessed using Kruskal-Wallis and Dunn’s post-test. A431 vaginal epithelial cells were challenged with *C*. *albicans* (0.01 MOI) for 24 h. Cell supernatants were assessed for **(E)** IL-1β release by ELISA (mean ± SD) and **(F)** LDH release (mean ± SD). Statistical significance was assessed using Student’s t-test. **(G)** Representative images of SC5314 and 529L during *in vivo* (vaginal lavage fluid) and *in vitro* (RPMI-1640) growth depicting filamentation (green arrows) and neutrophil recruitment (yellow arrows). **(H)** In vitro *ECE1* expression levels were assessed by qRT-PCR 4 h after strains were transferred to RPMI-1640 and normalized to *ACT1* expression values and SC5314 using the ΔΔCt method (mean ± SD). **(I)** In vivo *ECE1* expression was assessed by qRT-PCR on vaginal lavage samples obtained at d 3 post-infection and normalized to *ACT1* expression values and SC5314 using the ΔΔCt method (mean ± SD). Statistical significance was assessed using Student’s t-test. All in vitro experiments were conducted in biological triplicate. *, p < 0.05, ** p < 0.01, *** p < 0.001.

The established mouse model of VVC was utilized to investigate whether vaginal immunopathology elicited by *C*. *albicans* clinical isolate 529L differs from reference strain SC5314. Compared to strain SC5314, clinical isolate 529L exhibited similar colonization (**Fig 1A**) but elicited significantly lower polymorphonuclear leukocyte (PMN) recruitment (**Fig 1B**), release of the major NLRP3 inflammasome effector IL-1β (**Fig 1C**), and tissue damage (**Fig 1D**). Consistent with *in vivo* results, strain 529L showed significantly reduced IL-1β release (**Fig 1E**) and cellular damage (**Fig 1F**) during challenge of A431 vaginal epithelial cells. Both SC5314 and 529L formed hyphae during growth *in vivo* and *in vitro* (**Fig 1G**). As candidalysin drives neutrophil recruitment and immunopathology at the vaginal mucosa, *ECE1* gene expression levels of both strains were measured by qRT-PCR during growth in RPMI-1640 and during murine vaginal infection [11]. Surprisingly, no significant difference was identified between these isolates (**Fig 1H and 1I**). Collectively, *C*. *albicans* strain 529L elicits reduced vaginal immunopathology, independent of colonization, filamentation, and *ECE1* gene expression which were similar to strain SC5314.

### Strain 529L harbors an alternative candidalysin allele possibly contributing to reduced vaginal immunopathology

**Fig 2:**
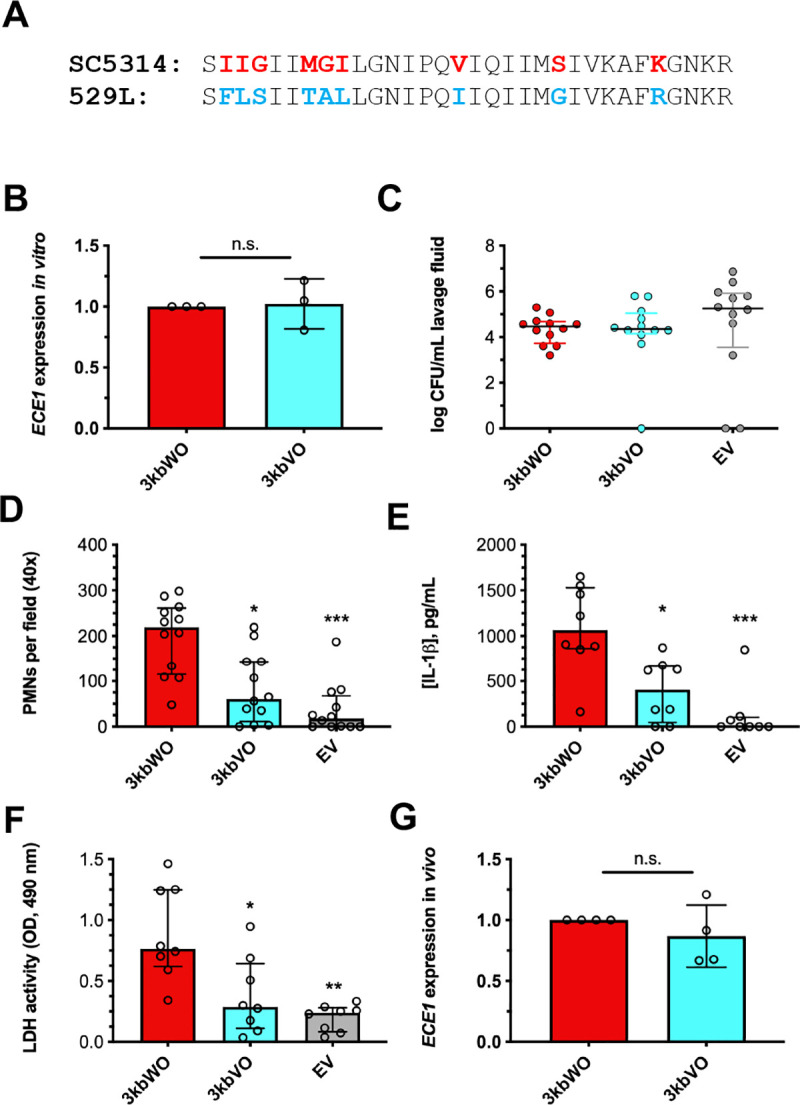
Isolate 529L harbors an alternative candidalysin allele. **(A)** Diagram depicting conserved (black), SC5314-like (red), 529L-like (blue) amino acids. **(B)** *In vitro ECE1* expression levels were assessed by qRT-PCR 4 h after strains were transferred to RPMI-1640 and normalized to *ACT1* and 3kbWO using the ΔΔCt method (mean ± SD). Statistical significance was assessed using a Student’s t-test. Mice (n = 12 per group) were challenged with 3kbWO, 3kbVO, and empty vector strains and vaginal lavage performed at d 3 post-infection. Lavage fluids were assessed for **(C)** CFU by microbiological plating (median ± IQR), **(D)** PMN recruitment by microscopy (median ± IQR), **(E)** IL-1β by ELISA (median ± IQR), and **(F)** tissue damage by LDH assay (median ± IQR). Statistical significance was assessed using Kruskal-Wallis and Dunn’s post-test. **(G)** *ECE1* expression (mean ± SD) was assessed *in vivo* by qRT-PCR as fold-change of *ECE1* over *ACT1* and normalized to 3kbWO using the ΔΔCt method. Significance was assessed using a Student’s t-test. All in vitro experiments were conducted in biological triplicate. *, p < 0.05, ** p < 0.01, *** p < 0.001.

Although *in vitro ECE1* gene expression levels were similar, sequencing analysis revealed variation in the nucleotide sequences encoding for candidalysin between *C*. *albicans* strains SC5314 and 529L (**Fig 2A**). Thus, to test the functional relevance of this variant, we constructed 3kbWO (wild-type ORF from SC5314) and 3kbVO (variant ORF from 529L) strains by transforming an isogenic *ece1*Δ/Δ strain with a vector containing approximately 3 kb of the 5’ untranslated region (UTR) through approximately 350 bp of the 3’UTR from strains SC5314 and 529L. Empty vector was also transformed into the *ece1*Δ/Δ strain. *ECE1* expression levels of 3kbWO and 3kbVO strains were equivalent *in vitro* as assessed by qRT-PCR (**Fig 2B**). Intravaginal challenge of mice with these strains revealed similar colonization (**Fig 2C**) and *ECE1* expression levels *in vivo* (**Fig 2G**). However, the 3kbVO strain elicited significantly lower PMN recruitment (**Fig 2D**), IL-1β release (**Fig 2E**), and tissue damage (**Fig 2F**), suggesting that discrepant *ECE1* alleles at least partially contributes to the reduced vaginal immunopathology elicited by strain 529L.

In order to account for promoter variability, isogenic strains TWO and TVO were constructed to express SC5314 and 529L *ECE1* alleles from the constitutive *TEF1* promoter respectively, in the same isogenic *ece1*Δ/Δ background. *In vitro ECE1* gene expression levels were measured by qRT-PCR, and *in vivo* vaginal immunopathology was assessed. *ECE1* expression levels and vaginal colonization were consistent among both strains (**S1A and S1B Fig**), yet challenge with TVO led to significantly reduced PMN recruitment (**S1C Fig**), IL-1β release (**S1D Fig**), and tissue damage **(S1E Fig)** as compared to TWO. Together, these data suggest that variation in *ECE1* coding sequences contributes to reduced vaginal immunopathology of strain 529L that is independent of gene expression.

### WT and variant candidalysin peptides exhibit similar capacity to induce damage, cytokine response, and membrane leakage

**Fig 3:**
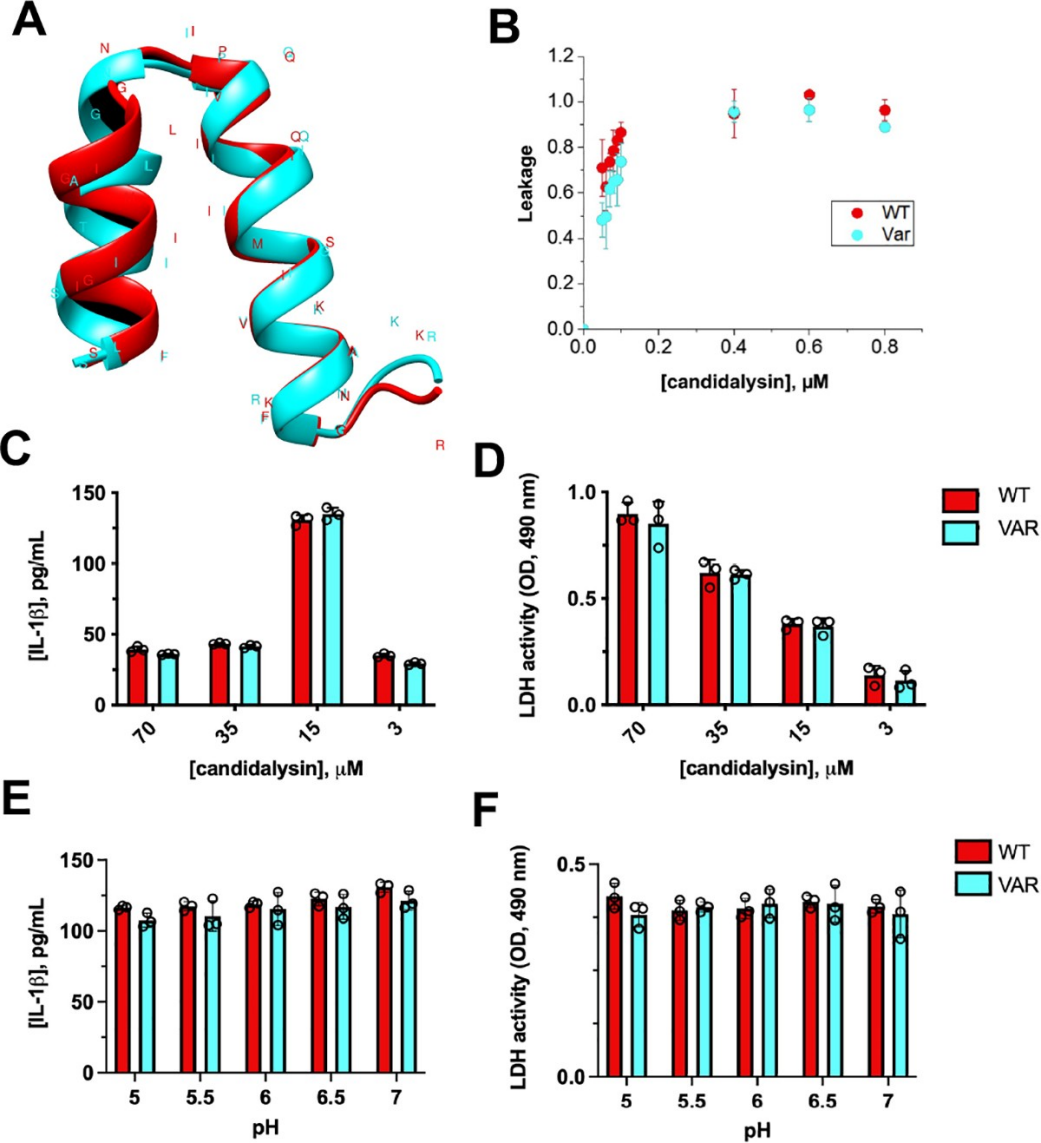
WT and variant candidalysin peptides similarly induce damage, cytokine response, and membrane leakage. **(A)** In silico modeling of candidalysin from both SC5314 and 529L reveal dihelical peptides with relatively conserved structure. **(B)** Sulforhodamine B (SRB) loaded lipid vesicles were treated with WT and VAR candidalysin for 1 h and fluorescence intensity (ex. 550/em. 590 nm) used to assess the capacity to induce membrane leakage (mean ± SD). A431 vaginal epithelial cells were challenged with wild-type (WT) or variant (VAR) candidalysin at doses of 70, 35, 15, and 3 μM and resulting **(C)** IL-1β and **(D)** LDH responses assessed (mean ± SD). Values were blank subtracted using vehicle-treated controls. A431 experiments were conducted as described above but candidalysins (15 μM) were added to buffered cell culture media adjusted to the indicated pH and **(E)** IL-1β (mean ± SD) and **(F)** LDH (mean ± SD) measured. All experiments were conducted in biological triplicate. Data was analyzed using multiple t-test.

*De novo in silico* modeling was performed to elucidate whether amino acid substitutions in SC5314- and 529L-like candidalysin were predicted to drastically alter peptide structure. Analysis revealed di-helical peptides with relatively conserved structure for both peptide isoforms (**Fig 3A**). In order to test whether the variant candidalysin contributed to observed phenotypic differences, synthetic wild-type (WT) and variant (VAR) candidalysins were used in a sulforhodamine B leakage assay. Surprisingly, treatment of vesicles with both candidalysin isoforms resulted in similar dose-dependent membrane leakage (**Fig 3B**). Consistent with this finding, challenge of A431 vaginal epithelial cells with these peptides led to comparable IL-1β release and cellular damage (**Fig 3C and 3D**). In order to determine whether candidalysins were differentially activate at more acidic pH representative of the human vaginal tract, cell culture medium was pH-adjusted prior to candidalysin challenge. Candidalysin activity between WT and VAR isoforms did not significantly differ across the pH range evaluated (**Fig 3E and 3F**). Thus, these results collectively suggested that the variant candidalysin peptide itself is capable of eliciting WT-like pathogenicity.

### The variant *ECE1* allele is associated with reduced candidalysin secretion

**Fig 4:**
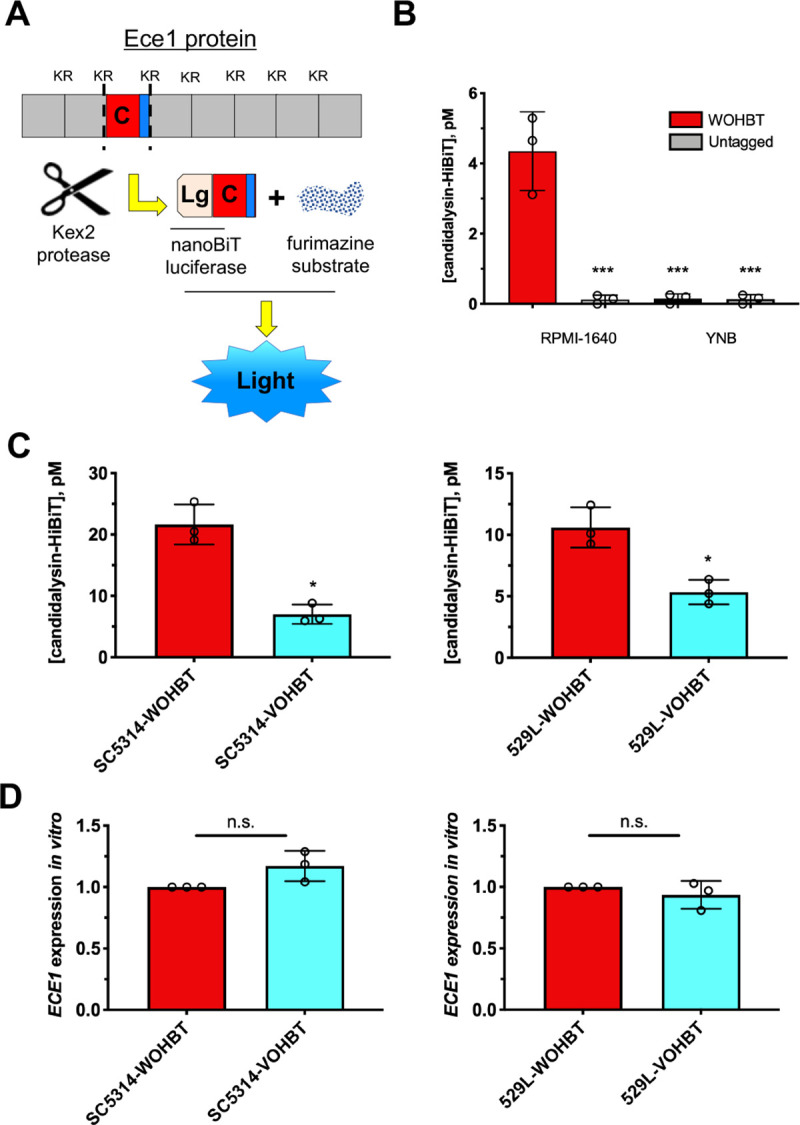
Variant *ECE1* is associated with reduced candidalysin secretion. **(A)** Schematic depicting C-terminal candidalysin HiBiT tag within the *ECE1* open reading frame and general assay function. **(B)** Strains GP1 (untagged, open bars) or WOHBT (HiBiT tagged, closed bars) were grown in RPMI-1640 or YNB medium for 16 h, supernatants harvested by centrifugation, and luminescence monitored using the Nano-GloⓇ Extracellular Detection System (mean ± SD). Statistical significance was assessed by one-way ANOVA and Dunnet’s post-test. **(C)** Secreted peptide was measured from SC5314 or 529L strains harboring WT or VAR HiBiT-tagged candidalysin as described above (mean ± SD). **(D)** *ECE1* expression levels were measured by qRT-PCR 4 h after transfer to RPMI-1640. Data were normalized to *ACT1* and WOHBT using the ΔΔCt method (mean ± SD). Statistical significance was assessed using Student’s t-test. All experiments were conducted in biological triplicate. *, p < 0.05, *** p < 0.001, n.s., not significant.

We next questioned whether candidalysin is less efficiently secreted in strain 529L. We applied the Nano-GloⓇ HiBiT Extracellular Detection System to quantify extracellular candidalysin secretion by HiBiT-tagging the 3’ end of the candidalysin coding sequence prior to the encoded C-terminal KR (**Fig 4A**) in the context of respective *ECE1* ORFs. Extracellular candidalysin concentration was monitored by luminescence in cell-free culture supernatants. Our approach was validated by demonstrating hypha-dependent, tag-specific detection of candidalysin in culture supernatants (**Fig 4B**). HiBiT tagged WT and variant *ECE1* ORFs were also introduced into SC5314 and 529L to monitor candidalysin secretion. Strikingly, both SC5314 and 529L strains secrete WT HiBiT-tagged candidalysin more efficiently than variant (**Fig 4C**), despite similar *ECE1* gene expression (**Fig 4D**). Collectively, these results revealed that the *ECE1* coding sequence may predict the capacity to which strains process and secrete candidalysin and may impact relative pathogenicity of *C*. *albicans*.

### The *ECE1* P2-candidalysin junction is critical for optimal pathogenicity

**Fig 5:**
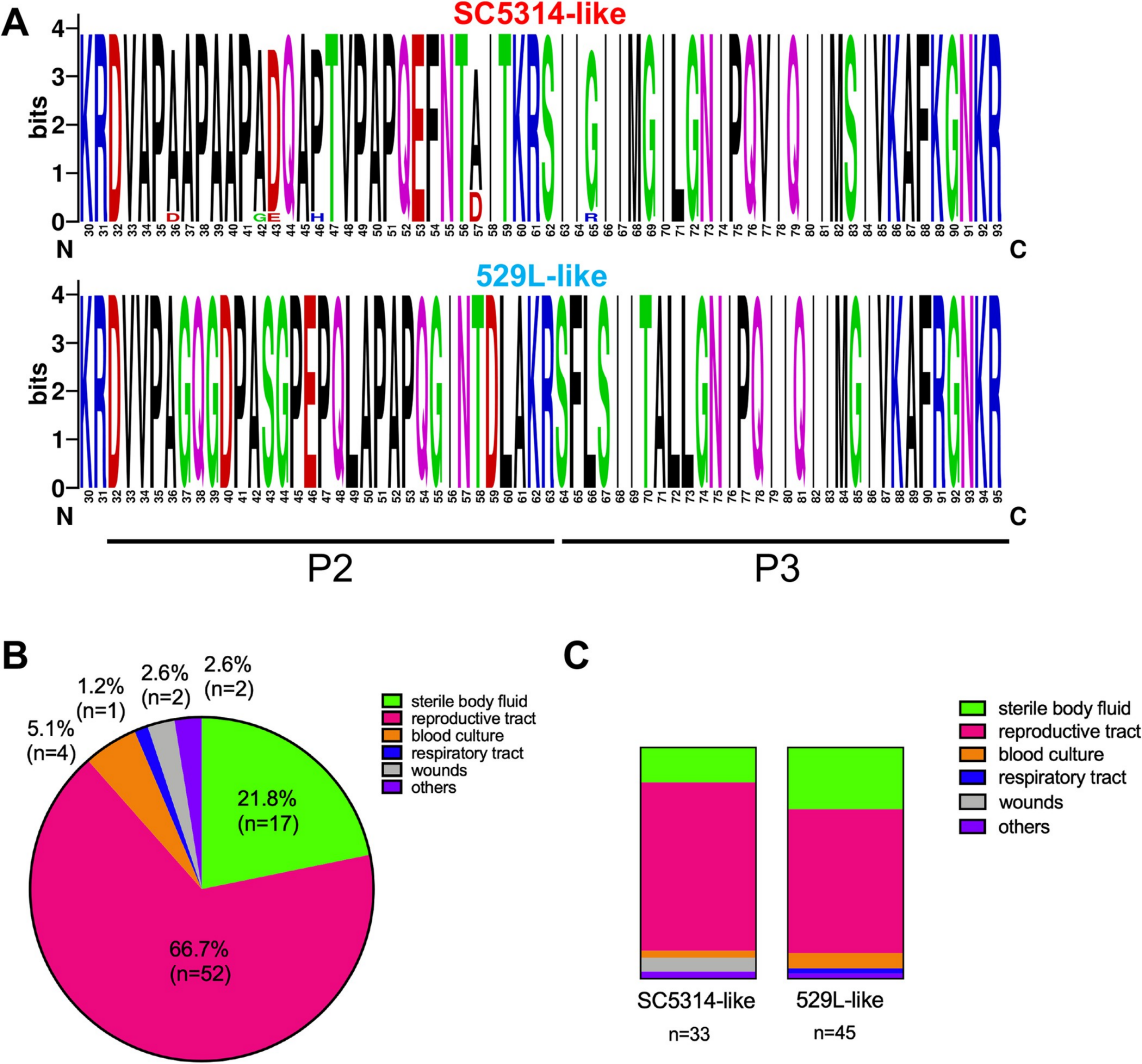
SC5314- and 529L-like candidalysin sequences reveal respective conserved *ECE1* P2 sequences. **(A)***ECE1* sequence data from S1 Table (n = 78 isolates) was analyzed using WebLogo software to generate a graphical representation of amino acids across *ECE1* P2 and P3 sequences from strains with SC5314- and 529L-like candidalysin sequences. Distribution of the isolates across **(B)** infection types and **(C)** SC5314 or 529L-likeness.

The Ece1p sequences in strains SC5314 and 529L were further compared and variants were identified in peptide 2 (P2) sequences besides those previously shown for candidalysin (P3). In order to examine broader *ECE1* P2-P3 sequence variation, genomic DNA from 78 global clinical isolates were amplified and PCR products subjected to Sanger sequencing. The sequences were aligned, grouped as SC5314- and 529L-like (**S4 Table**), and graphical sequence alignments generated (**Fig 5A**). Strikingly, a high level of conservation was noted in *ECE1* P2 regions that correlated with either SC5314- or 529L-like candidalysin sequences; all strains with a variant candidalysin also harbored this alternate P2 sequence. A majority of the isolates analyzed were obtained from the female reproductive tract or sterile body fluid (**Fig 5B**). Interestingly, slightly more strains harbored the 529-like *ECE1* allele but the distribution of each did not appear to be correlated to anatomical site (**Fig 5C**).

**Fig 6:**
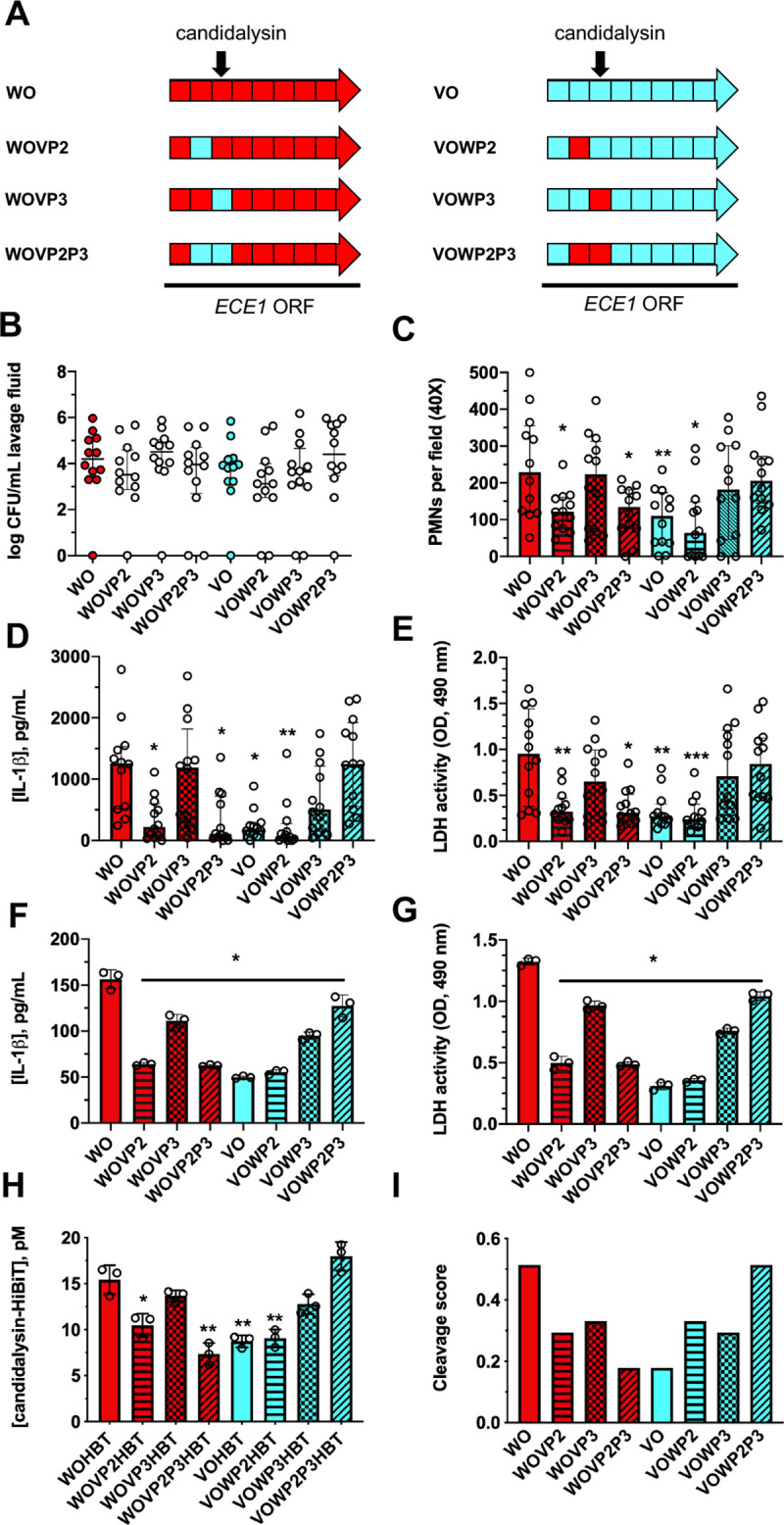
The *ECE1* P2-P3 sequence combination is critical for peak immunopathology and candidalysin secretion. **(A)** Schematic showing *ECE1* open reading frames containing SC5314 or 529L P2, P3, or P2-P3 sequences that were restored in an isogenic *ece1*Δ/Δ strain. Strains were intravaginally inoculated into estrogen-treated C57BL/6 mice. Mice (n = 12 per group) underwent vaginal lavage at d 3 post-infection and lavage fluids assessed for **(B)** CFU by microbiological plating (median ± IQR), **(C)** PMN recruitment by microscopy (median ± IQR), **(D)** IL-1β by ELISA (median ± IQR), and **(E)** tissue damage by LDH assay (median ± IQR). Statistical significance was assessed using Kruskal-Wallis and Dunn’s post-test. A431 vaginal epithelial cells were challenged with these same strains and **(F)** IL-1β measured by ELISA (mean ± SD) and **(G)** LDH release assessed (mean ± SD). **(H)** Similar strains harboring C-terminal candidalysin HiBiT tags were grown overnight in RPMI-1640 medium and cell-free supernatants assessed for luciferase activity (mean ± SD). Statistical significance was assessed by one-way ANOVA and Dunnet’s post-test. **(I)** P2-P3 sequences were parsed using ProP 1.0 Server to determine cleavage scores. All in vitro experiments were conducted in biological triplicate. *, p < 0.05, ** p < 0.01, *** p < 0.001.

In order to determine the contribution of *ECE1* P2 or P3 to the relative pathogenicity of *C*. *albicans*, *ECE1* ORFs containing WT or variant P2, P3, or P2-P3 sequences were restored in an isogenic *ece1*Δ/Δ background derived from strain BWP17 which descended from SC5314 (**Fig 6A**). All strains demonstrated similar *ECE1* gene expression (**S2A Fig**), growth (**S2C Fig**), and hypha formation (**S2D Fig**). Also, all strains colonized similarly (**Fig 5B**) but with varying immunopathogenicity when intravaginally inoculated into mice. Strains reconstituted with WT *ECE1* ORF background (WO), WOVP2 (variant P2) and WOVP2P3 (variant P2-P3) showed significantly lower pathogenicity (PMN recruitment, IL-1β, and tissue damage) than WO (**Fig 6C, 6D and 6E**). WOVP3 containing variant P3 displayed similar pathogenicity as WO, revealing the importance of WT *ECE1* P2 in driving candidalysin-associated virulence. In strains containing a variant *ECE1* ORF background (VO), the pathogenicity of VOWP2 (WT P2) were similar to VO, which was significantly lower than WO. However, VOWP2P3 (WT P2-P3) restored pathogenicity to the same level of WO, suggesting the combination of WT *ECE1* P2-P3 sequence is sufficient to induce WT-level vaginal immunopathogenesis (**Fig 6B, 6C, 6D and 6E**). Challenge of A431 vaginal epithelial cells with these strains led to similar patterns with respect to IL-1β (**Fig 6F**) and LDH release (**Fig 6G**), further confirming the *ECE1* P2-P3 sequence combination is critical for peak toxin-associated virulence.

The processing of Ece1p relies on the cleavage by Kex2p, which identifies KR repeats as peptide substrates [13]. Prior work showed that Kex2 substrates are processed with varying efficiency depending on the amino acids that immediately flank the N- and C-terminal ends of the KR cleavage site [17]. Thus, the observed variation in P2-P3 may result in lower cleavage efficiency. A similar HiBiT-tagging approach was utilized to assess candidalysin secretion levels in these chimeric strains. Despite similar *ECE1* gene expression in all strains (**S2B Fig**), only strains harboring the WT *ECE1* P2-P3 sequence exhibited WT level candidalysin secretion, suggesting that the combination of WT P2 and P3 leads to the most efficient candidalysin processing (**Fig 6H**). Moreover, release of HiBiT-tagged candidalysin into the culture medium by each chimeric strain was positively correlated to IL-1β release and cell damage as assessed by linear regression analyses (**S3A and S3B Fig**). In addition, sequences parsed with ProP 1.0 Server, a Kex2-like substrate prediction tool, revealed that the sequence TAITKRSI from SC5314 yields a higher score (0.524) than TDLAKRSF from 529L (0.179) (**Fig 6I**) supporting the experimental HiBiT-tagging data [21].

### Expression of the SC5314 *ECE1* locus in strain 529L partially restores pathogenicity

**Fig 7:**
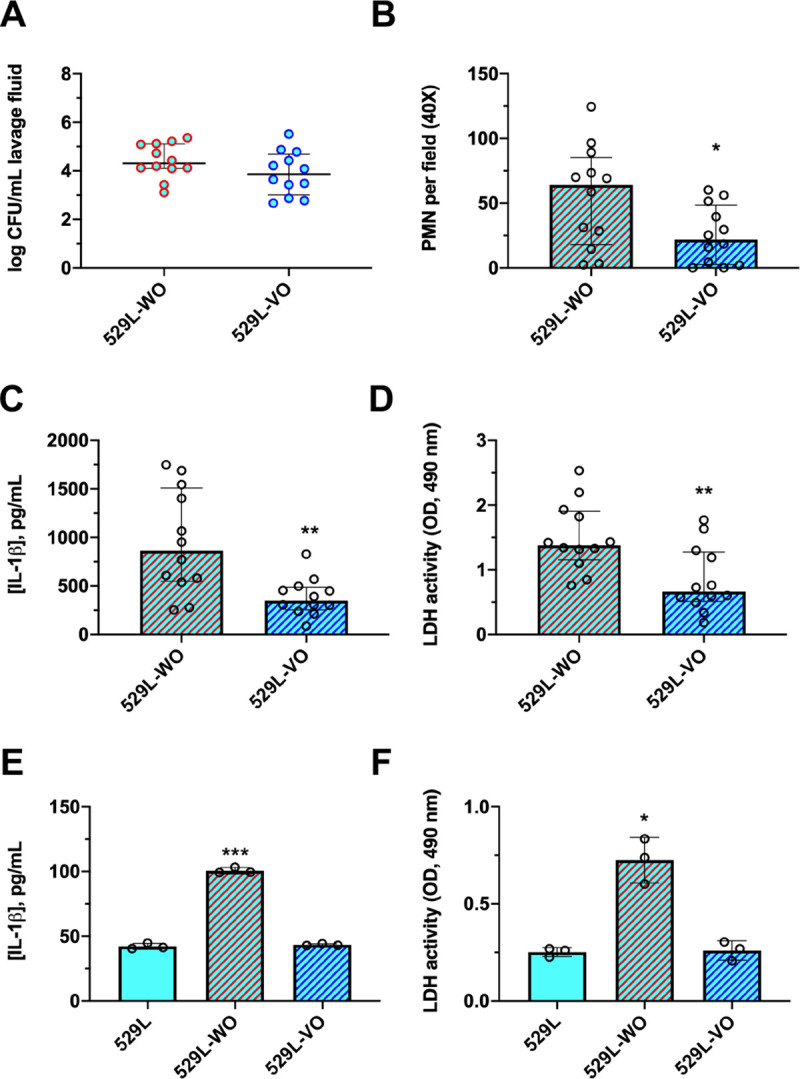
Introduction of the SC5314 *ECE1* locus in isolate 529L partially restores pathogenicity. Mice were challenged with strain 529L harboring SC5314 *ECE1* (529L-WO) or an additional copy of 529L *ECE1* (529L-VO). Mice (n = 12 per group) underwent vaginal lavage at d 3 post-infection and lavage fluids assessed for **(A)** CFU by microbiological plating (median ± IQR), **(B)** PMN recruitment by microscopy (median ± IQR), **(C)** IL-1β by ELISA (median ± IQR), and **(D)** tissue damage by LDH assay (median ± IQR). Statistical significance was assessed using Mann-Whitney U test. A431 vaginal epithelial cells were challenged with these same strains, along with isolate 529L, and **(E)** IL-1β measured by ELISA (mean ± SD) and **(F)** LDH release (mean ± SD) assessed. Statistical significance was assessed using one-way ANOVA and Dunnet’s post-test. All in vitro experiments were conducted in biological triplicate. *, p < 0.05, ** p < 0.01, *** p < 0.001.

Finally, we investigated whether the acquisition of a WT *ECE1* allele by strain 529L could restore WT-level pathogenicity. A single copy of either WT or variant *ECE1* allele was transformed into strain 529L to construct strains 529L-WO and 529L-VO. The two strains were intravaginally inoculated into mice and assessed for pathogenicity. Despite similar colonization (**Fig 7A**) immunopathogenicity was notably different. PMN recruitment (**Fig 7B**), IL-1β (**Fig 7C**), and tissue damage (**Fig 7D**) in mice challenged with strain 529L-WO were significantly higher than strain 529L-VO. Thus, strain 529L-WO elicited higher vaginal immunopathology than strain 529L-VO. In addition, A431 vaginal epithelial cells were challenged with the aforementioned strains and 529L. IL-1β release and cellular damage caused by strain 529L-WO were significantly higher than those of strains 529L and 529L-VO (**Fig 7E and 7F**). Hence, introduction of the WT *ECE1* allele into strain 529L partially restores pathogenicity, presumably due to more efficient candidalysin secretion (**Fig 4D**).

## Discussion

Pathogenicity mechanisms of *C*. *albicans* are largely viewed through the lens of the reference isolate SC5314. However, intraspecies heterogeneity is highly relevant to strain-dependent pathogenicity and the virulence and fitness mechanisms employed by natural isolates is underappreciated [22,23,24]. One recent study demonstrated that despite all isolates retaining the capacity to invade murine oral epithelium, infection with SC5314 resulted in generally higher and more consistently increased host innate immune cytokine responses, neutrophil recruitment, and mucosal damage [25]. Similar to results reported here, challenge with 529L led to comparable (or elevated) early colonization of the tongue, yet markers of immune activation and damage were significantly decreased, despite similar levels of *ECE1* and *KEX2* gene expression. While 529L was capable of delayed innate cytokine induction, this was not associated with neutrophil migration, suggesting that these events are uncoupled in the murine oral cavity. Strain 529L is reported to exhibit decreased hyphal length and increased pseudohyphal growth which may impact its virulence [19,25]. That said, Roselletti and colleagues recently demonstrated that pseudohyphal growth of *C*. *albicans* vaginal isolates was associated with elevated IL-1β release and high expression of multiple inflammasome activators, including *ECE1* [26]. Moreover, we routinely observed elongated hyphal filaments formed by 529L both in vitro and in vivo (**Fig 1G**) suggesting that disparately reported hyphal growth dynamics may be anatomical site-, medium- or time-dependent. Collectively, our observations at the murine vaginal mucosa support these prior results, demonstrating that the capacity to induce damage is linked to neutrophil migration and that *ECE1* expression alone does not serve as a surrogate marker of virulence, as inefficient candidalysin secretion would likely impact pathogenicity. Allelic diversity at this locus is just a single variable that should be considered with respect to isolate-specific virulence—metabolic plasticity, capacity to form hyphae, general growth rate, and expression of other secreted virulence determinants are equally relevant factors.

Although we have only characterized a single *ECE1* allelic variant derived from the strain 529L, these results may be broadly relevant across clinical isolates. A survey of *ECE1* P2-P3 sequences from *C*. *albicans* isolates from a variety of biological sites and across two continents revealed relatively conserved predicted amino acid sequences that fall into two major groups: those that are similar to SC5314 and those similar to 529L (**Fig 5**). Out of the 78 isolates sequenced, ~42% aligned closely to the SC5314-like sequence. Distribution of each isoform at various biological sites was similar, suggesting no clear advantageous role for a given allele. Unfortunately, additional information as to whether a majority of these isolates were associated with active disease or colonization is unknown. Given our results, it is reasonable to speculate that a 529L-like sequence may be associated with less pathogenicity, but such isolates were moderately over-represented in sterile body fluid and blood culture samples, suggesting otherwise. Intriguing evolution-related questions regarding how natural isolates acquire alternate *ECE1* alleles, which selective pressures drive their maintenance, how such differences tip the balance of commensal versus pathogen status, and global distribution across infections remain.

The majority of isolates obtained were indeed from the female reproductive tract. While the mouse model of VVC has been an indispensable tool to dissect host immune mechanisms associated with disease, disparate microbiological, physiological, and biochemical landscapes of the vaginal tract exist between mouse and human. One notable difference is the near neutral vaginal pH of mice (pH 6.5–7) which contrasts with a much more acidic vaginal environment observed in women (pH 3.5–5.0) [27]. Acidic pH is known to impair *C*. *albicans* hyphal growth and thus logically also *ECE1* expression [28]. However, the pH of the human vagina does not alkalinize during symptomatic VVC, suggesting that *C*. *albicans* is able to overcome this repressive condition to form hyphae and invade the vaginal mucosa [29]. Perhaps this is due to the capacity of *C*. *albicans* to alkalinize its external environment through amino acid degradation and secretion of ammonia [30]. In any case, results in **Fig 3E and 3F** suggest that both WT and variant candidalysin peptides are equally active across an increasingly acidic pH range and offers translatability of the importance of candidalysin-mediated damage and our collective findings to the setting of human VVC.

It is clear from our results that introduction of the 529L *ECE1* allele as compared to SC5314 *ECE1* allele into an SC5314-based Δ/Δ*ece1* background reduces pathogenicity (**Fig 6**) and that introduction of an SC5314 *ECE1* allele into 529L (**Fig 7**) increases corresponding inflammatory markers, neutrophil migration, and damage. However, it is important to note that pathogenicity and candidalysin secretion is only reduced and not completely abrogated or restored to SC5314-like levels in these strains. Thus, while context of the *ECE1* coding sequence certainly contributes, it likely does not itself drive overall strain pathogenicity, for some of the reasons given above. A litany of altered signaling events, cell surface structures, and virulence determinants in 529L are likely relevant [31,32,33]. Until subsequent work is done to phenotype these isolates more extensively, contextual role of *ECE1* should be considered on a strain-to-strain basis.

Given results obtained from our isogenic strain construction approach, it was initially surprising to find that cytokine and damage biomarkers from vaginal epithelial cells did not differ between treatment with WT or variant candidalysin peptide. While candidalysin has been recently shown to accumulate in the “invasion pocket” during hyphal invasion of oral epithelium, the actual quantity of candidalysin released into the external environment or locally is not currently known [13,34]. Therefore, we cannot rule out the possibility that potentially supraphysiologic concentrations of these candidalysins used in cell culture assays may not show differential activity, but the distinction could become more obvious at natural levels released by *C*. *albicans* during infection. As a proxy of candidalysin secretion, we utilized a HiBiT-tagging approach where a short sequence coding for a small domain of Nano-luciferase was fused in-frame with the C-terminal end of candidalysin and, to our knowledge, this is the first time this system has been applied to study protein secretion in fungi [35]. Despite the addition of this C-terminal tag and potential complications with Kex2 processing, secretion levels largely mirrored host inflammatory and damage biomarkers of untagged versions both in vitro and in vivo. While “candidalysin” release was quantitated in the picomolar range in such assays, this was in fact a fusion peptide and may not definitively reflect secretion levels of native candidalysin. However, the approach should still be applicable for studying the relative processing and secretion of multiple *C*. *albicans* peptide substrates, including other virulence determinants.

Except for peptide 3 (P3, i.e. candidalysin), the function of the 7 other peptides remain unknown, but our results suggest that at least P2 may play a key structural role for efficient processing of the Ece1p polypeptide. A prior study revealed that efficient cleavage of KR residues at the terminal ends of peptide 2 and 3 (P2 and P3) by the Kex2p protease are necessary for toxin activity on oral epithelial cells and for full virulence in a mouse model of OPC [13,18]. Interestingly, the majority of genetic diversity at the *ECE1* locus falls within regions encoding for these peptides. Previous work demonstrated that amino acid sequences immediately flanking the KR sites alters substrate cleavage efficiency by Kex2p [17]. Based on a position-specific scoring matrix (curated as p4-p3-K-R) used to identify potential Kex2 substrate proteins, SC5314-like scores (p4: I = 0.28, p3: T = 0.77) are higher than those of 529L-like sequences (p4: L = 0.09, p3: A = 0.64), similar to our predicted furin cleavage results. It was noted that predicted cleavage scores (**Fig 6I**) did not perfectly correlate with HiBiT-tagged candidalysin secretion (**Fig 6H**). For example, VOWP2 and VOWP3 sequences yield very similar cleavage scores, yet levels of secretion, damage, and IL-1β processing are markedly disparate. Additionally, it is tempting to ascribe necessary and sufficient roles of individual Ece1p peptides for pathogenic activity but interpretation remains speculative. As for the SC5314-like *ECE1* allele, exchange of P2 with the variant sequence (WOVP2) is sufficient for impairing toxin secretion (**Fig 6**). However, introduction of the SC5314-like P3 sequence in the 529L-like allele (VOWP3) increases candidalysin secretion and pathogenicity despite this strain still harboring a variant P2 sequence. Thus, necessary and sufficient roles appear dependent on allelic context and cannot be broadly defined. However, it is clear that an SC5314-like continuous P2-P3 sequence (WO or VOWP2P3) is sufficient for achieving maximal pathogenicity perhaps due to preferential Kex2p cleavage sites flanking the junction of these peptides (**Fig 6**). Alterations in overall protein structure imparted by disparate Ece1p peptides may affect polypeptide folding, stability, or contact with Kex2p active sites and these effects may partly explain discordant results in chimeric strains. Biochemical assays and site directed mutagenesis approaches to conclusively address differential cleavage are currently underway.

In summary, this study demonstrates that genetic variation at the *ECE1* locus contributes to reduced pathogenicity of *C*. *albicans* clinical isolate 529L during vulvovaginal candidiasis. Furthermore, the 529L-like P2-candidalysin sequence is represented in numerous clinical isolates and has reduced capacity to elicit neutrophils, IL-1β, and damage, at least partially due to impaired secretion. The findings in this study identify a novel mechanism for restrained candidalysin activity which may impact commensalism, fitness, and pathogenicity at the mucosal surface. Furthermore, genotyping of *ECE1* alleles across clinical isolates may inform insight into the evolution of virulence in this exceedingly common opportunistic fungus.

## Materials and methods

### Ethics statement

The animals used in this study were housed in AAALAC-approved facilities located in the Regional Biocontainment Laboratory (RBL) at the University of Tennessee Health Sciences Center (UTHSC). The UTHSC Animal Care and Use Committee approved all animals and protocols (protocol number 18–065) in accordance with the Guide for the Care and Use of Laboratory Animals. Mice were given standard rodent chow and water ad libitum. Mice were monitored for signs of distress, including noticeable weight loss and lethargy. Mice were anesthetized as needed with 3% isoflurane in oxygen and sacrificed by isoflurane overdose followed by cervical dislocation in accordance with UTHSC IACUC guidelines.

### Microorganism growth

*C*. *albicans* strains were maintained as glycerol stocks stored at −80°C. A small amount of stock was spread onto yeast extract-peptone-dextrose (YPD) agar and incubated at 30°C for 48 h to obtain isolated colonies. A single colony was transferred to 5 ml of YPD and incubated at 30°C with shaking at 200 rpm for 18 h prior to vaginal infection. Transformant selection was performed on minimal YNB medium (6.8 g/L yeast nitrogen base without amino acids, 0.5% dextrose, 2% Bacto agar) supplemented with the appropriate auxotrophic requirements 100 μg/mL L-histidine or L-arginine or 50 μg/mL uridine as previously described [36,37]. Cloning vectors were maintained in *Escherichia coli* DH5⍺ and positive transformants selected for growth in Luria-Bertani medium containing 100 μg/mL ampicillin.

### Strains and primers

All strains used or generated for construction of promoter replacement, *ECE1* alleles, and *ECE1* peptide swaps and controls can be found in **S1 Table**. All primers used for strain construction or quantitative PCR (qPCR) are listed in **S2 Table** in the supplemental material. All gBlock sequences used for construction of *ECE1* ORF and peptide swap strains can be found in **S3 Table**.

### Vector construction

In order to generate strains harboring approximately 3 kb of the native *ECE1* promoter, entire open reading frame (ORF), and 350 bp of the terminator, PCR products were amplified from genomic DNA isolated from *C*. *albicans* strains SC5314 and 529L using primers ECE1Pr3k-F-StuI and tECE1-R-SacI and SuperFi high fidelity polymerase. Amplicons were digested with StuI and SacI prior to ligation into StuI+SacI digested pLUX [38]. In order to generate *ECE1* ORFs driven by the *TEF1* promoter, PCR products were generated by amplification of genomic DNA with SuperFi high-fidelity polymerase using primers ECE1ORFF-SalI and ECE1ORFR-MluI. Amplicons were digested with SalI and MluI prior to ligation into SalI+MluI digested pKE4 [39]. In order to generate a plasmid harboring the SC5314 *ECE1* promoter, vector pKE4-GFPy was digested with StuI and SalI and a similarly digested PCR product generated using primers ECE1Pr3k-F-StuI and PrECE1-R-SalI cloned into that site to yield plasmid Pr*ECE1*-GFPy [27]. In order to generate *ECE1* peptide swap strains driven by a common SC5314 *ECE1* promoter, gBlocks (Integrated DNA Technologies) were cloned into vector Pr*ECE1*-GFPy by digestion with SalI and MluI to replace the GFPy coding sequence. Plasmids pKE4 and Pr*ECE1*-GFPy contain an *ADH1* terminator sequence, while pLUX contains the native terminator. All plasmids contain a full length *URA3* locus, and target integration at native *IRO1*-*URA3* loci restoring both *IRO1* function and *URA3* prototrophy. Lastly, vector pDUP3 (encoding for nourseothricin resistance and targeted integration at the *NEUT5L* locus) was digested with ClaI and SpeI [40]. PCR products were generated from amplification of linearized plasmids containing PrECE1-ECE1-tADH1 or Pr*ECE1*-*ECE1*HBT-t*ADH1* sequences using primers PrECE1-F-ClaI and AHDtermv2-R-SpeI, digested with ClaI and SpeI, and cloned into pDUP3. All plasmids were verified for correct coding sequence by Sanger methodology (Genewiz) using primers ECE1ProSEQ4F and ADH13SEQR or TEF1PRSEQF and ADH13SEQR.

### Strain construction

*ECE1* deletion cassettes were amplified by PCR with primers ECE1DISF and ECE1DISR using pRS-ARG4ΔSpeI or pGEM-HIS1 as templates. The *ece1*Δ/Δ *ura3*Δ/Δ deletion mutant was produced by sequential deletion of each *ECE1* allele using *HIS1* and *ARG4* markers and transformation by the lithium acetate method and plating on selective media as described [13]. Correct integration of deletion cassettes was confirmed at each step by PCR of genomic DNA with primer pairs ARG4INTF2/ECE1AMPR and ARG4INTR2/ECE1AMPF (ARG4 integration), or HIS1INTR2/ECE1AMPR and HIS1INTF2/ECE1AMPF (HIS1 integration). Lack of an intact *ECE1* allele was confirmed by using primer pair ECE1DETF and ECE1DETR. An isogenic *ece1*Δ/Δ mutant (EV) and *ECE1*-reconstituted strains were produced by transforming the *ece1*Δ/Δ *ura3*Δ/Δ mutant with the following NheI-digested plasmids: pLUX (vector alone), pLUX-Pr3kbSCECE1, and pLUX-Pr3kb529LECE1 (native promoter constructs); pKE4-ECE1SC5314 and pKE4-ECE1529L (*TEF1* promoter constructs), or Pr*ECE1*-*ECE1*-t*ADH1* containing ORFs, *ECE1* peptide swaps, or HiBiT-tagged alleles (gBlock constructs, **S3 Table**). Correct integration of plasmids at the *IRO1*-*URA3* locus was confirmed by PCR using primer pair LUXINTDETF/LUXINTDETR and restoration of *ECE1* verified using primer pairs ECE1DETF/ECE1DETR. In order to create wild-type SC5314 or 529L strains harboring untagged or HiBiT-tagged *ECE1* alleles, pDUP3-based plasmids were digested with SfiI and transformed into the relevant *C*. *albicans* strain by lithium acetate method as described [41]. Transformants were selected by plating onto YPD containing 200 μg/mL nourseothricin. Integration at the *NEUT5* locus was confirmed using primer pairs NEUT5LAMPF and NAT1INTF and NEUT5LAMPR and ECE1invarQPCR-R.

### Murine model of vulvovaginal candidiasis

All experiments were approved by the UTHSC Animal Care and Use Committee and are consistent with the NIH Guide for the Care and Use of Laboratory Animals. The murine model of vulvovaginal candidiasis has been extensively reported in the literature and was performed as described previously [10]. Female 6- to 8-week old C57/BL6 mice were purchased from Charles River Laboratories and housed in isolator cages mounted on ventilator racks. Mice were subcutaneously administered 0.1 mg of β-estradiol 17 valerate (Sigma) dissolved in 0.1 mL of sesame oil 72 h prior to inoculation with *C*. *albicans*. Stationary phase cultures of *C*. *albicans* were washed 3X with sterile phosphate-buffered saline (PBS) and resuspended in 0.2X volume of PBS. Cell suspensions were counted, enumerated using a hemocytometer, and adjusted to 5x10^8^ CFU/mL. Mice were intravaginally inoculated with 10 μL (5x10^6^ CFU) of the standardized cell suspension. Mice underwent vaginal lavage at d 3 post-inoculation (p.i.) with 100 μL PBS. Recovered vaginal lavage fluids (VLF) were spiked with 100X EDTA-free protease inhibitors (cOmplete; Roche). Samples were either used immediately or stored at -80°C. All animal experiments were conducted at least in duplicate (group size n = 4) and the resulting data combined.

### Assessment of fungal burden, immunopathology, and damage

Immunopathological markers were assessed as described previously [10]. Lavage fluid was serially diluted 10-fold using the drop-plate method and plated onto YPD agar containing 50 μg/mL chloramphenicol. Colonies were enumerated following 24 h incubation at 37°C. Lavage fluid (10 μL) was smeared onto glass slides and stained by the Papanicolaou method to assess PMN recruitment. Murine IL-1β was measured in clarified, diluted VLF by using a commercial enzyme-linked immunosorbent assay (ELISA, Thermo Fisher). Markers of epithelial damage were measured in VLF by a lactate dehydrogenase release assay (CytoTox 96 nonradioactive kit; Promega).

### General growth assay

*C*. *albicans* cultures were adjusted to 1x10^6^ cells/mL in YPD, added to wells of a microtiter plate, and optical density (OD) values at 600 nm collected kinetically using a Biotek Synergy H1 instrument set at 30°C incubation temperature with orbital shaking.

### Hyphal growth assay

*C*. *albicans* cultures were grown overnight in YPD medium, washed 3X in PBS, counted, and diluted to a density of 5x10^7^ cells/mL. Roswell Park Memorial Institute-1640 (RPMI), pH 7.0 containing 165 mM MOPS buffer medium was inoculated with 10 μL of adjusted cells suspension and incubated at 37°C with shaking at 200 rpm for 4 or 16 h, cultures imaged by standard light microscopy (Nikon Ni-U), and captured digitally.

### qRT-PCR analysis

RNA was extracted from *in vitro* and *in vivo* samples as described previously using the hot acid-phenol method [42]. For *in vitro* samples, overnight cultures of *C*. *albicans* grown in YPD were washed 3X in PBS and diluted 1:100 in fresh RPMI-1640. RNA was extracted after shaking (200 rpm) at 37°C for 4 h. RNA isolated from in vivo vaginal lavage material was assessed at d 3 p.i.. Extracted RNA concentrations were equalized among samples, and 200-ng aliquots were treated with RNase-free DNase according to the manufacturer’s instructions (Thermo Fisher). RNA was reverse transcribed using random hexamers and a RevertAid kit according to the manufacturer’s protocol (Thermo Fisher). Strain optimized forward and reverse primers (final concentration, 0.5 μM) for *ECE1* or *ACT1* were used in conjunction with 2X Maxima SYBR green according to the manufacturer’s instructions (Thermo Fisher) to amplify 100-bp fragments from approximately 20 ng of cDNA (**S2 Table**). qPCRs were monitored and analyzed with an Applied Biosystems 7500 platform and software. Expression levels of *ECE1* were compared to those of a reference gene (*ACT1*) and relevant control strain by using the ΔΔC_T_ method [43].

### Candidalysin challenge of epithelial cells

The A431 human vulvar epidermoid carcinoma cell line was utilized as described [11]. A431 cells were cultured in RPMI medium plus L-glutamine (Life Technologies) supplemented with 10% (vol/vol) heat-inactivated fetal bovine serum (Life Technologies) at 37°C and 5% CO_2_. Cells were seeded in 96 well plates at a density of 1x10^5^ cells/wells. Candidalysin peptide (WT: SIIGIIMGILGNIPQVIQIIMSIVKAFKGNK; VAR: SFLSIITALLGNIPQIIQIIMGIVKAFRGNK) was purchased from Peptide Protein Research Ltd. (United Kingdom). Prior to candidalysin challenge, confluent A431 epithelial cells were serum starved overnight, and all experiments were carried out in serum and phenol-red free RPMI. The cells were incubated with candidalysin (prepared as a 10-mg/ml stock in sterile water) at doses of 3, 15, 35, or 70 μM for 24 h at 37°C in 5% CO_2_ for 24 h. Sterile-water (vehicle-only) controls were also included. In some experiments, buffered cell culture medium was adjusted to various pH levels as noted. Cell supernatants were isolated and human IL-1β measured using a commercial ELISA (Thermo Fisher). Supernatants were also assessed for damage by using the LDH release assay described above.

### In silico modeling of candidalysin

Candidalysin sequences were parsed using QUARK (https://zhanglab.ccmb.med.umich.edu/services/) to generate ab initio predicted structural models [44]. Resultant PDB files were further annotated and formatted using UCSF Chimera software [45].

### Epithelial cell line *C*. *albicans* infection model

A431 vaginal epithelial cells were grown as described above and challenged with *C*. *albicans* strains (MOI: 0.01) for 24 h. Isolated supernatants were assessed for IL-1β and LDH release as described previously [11].

### Sulforhodamine B leakage assay

Fluorescence dequenching experiments (n = 3 independent replicates) compared the effectiveness of variant and wild-type candidalysin at inducing leakage of the sulforhodamine B (SRB) dye from lipid vesicles. Stocks of POPC:POPE:cholesterol (molar ratio 42:25:33, Avanti Polar Lipids, Inc., Alabaster, AL) in chloroform were dried in a glass tube under vacuum for >2 hours to create a thin lipid layer. To encapsulate the SRB, dried lipid was resuspended at 10 mM sodium acetate (pH 5.5) buffer containing 20 mM SRB. Large unilamellar vesicles (LUVs) were formed by extruding 100 times through a 200-nm polycarbonate filter (Whatman, Maidstone, United Kingdom) using a Mini-Extruder (Avanti Polar Lipids, Inc., Alabaster, AL). Free dye was separated from lipid-encapsulated dye using a PD-10 desalting column (GE Healthcare Life Sciences, Marlborough, MA) [46]. For measurement, lipid-encapsulated dye (50 μL, initially 259.2 μM), peptide (varying volumes, initially 10 μM) and buffer (varying volumes) were transferred to the wells of a 96-well plate for a final lipid concentration of 129.6 μM, and final peptide concentrations ranging from 0.05 to 0.8 μM. Alternatively, buffer or Triton X-100 detergent (final concentration was 0.016% by volume) replaced the peptide treatment to serve as negative and positive controls for leakage, respectively. After a one-hour incubation at room temperature and a five-second shake at a frequency of 567 cpm, fluorescence intensities were measured at 25.0°C using extended gain mode in a Cytation 5 microplate reader (BioTek Instruments, Inc., Winooski, VT) with a monochromator-filtered excitation wavelength of 550/9 nm and emission wavelength of 590/9 nm.

Data were corrected to account for the effect of TX-100 on the fluorescence intensity of SRB. This correction factor was determined for SRB in solution, and applied to encapsulated SRB samples. Leakage was calculated for each peptide concentration according to the following equation:

$$Leakage=\frac{\left(F_{pep}+F_{PBS}\right)}{\left(F_{TX}-F_{PBS}\right)},$$ (1)

where *F_pep_, F_PBS_*, and *F_TX_* are the fluorescence intensities of encapsulated dye after incubating with peptide, PBS, or Triton X-100, respectively.

### HiBiT-tagged candidalysin secretion assay

As a proxy to measure extracellular toxin release, *C*. *albicans* strains were engineered to express a HiBiT-tag at the C-terminus of candidalysin. The 11 amino acid HiBiT tag encodes for a small domain of NanoLucⓇ luciferase (Promega). *C*. *albicans* cells were grown in yeast nitrogen base (YNB) O/N, washed 3X in PBS, counted, diluted to a final density of 5x10^5^ cells/mL in MOPS-buffered RPMI-1640, and incubated at 37°C with shaking (200 rpm) for 16 h. The following day, cultures were centrifuged at high speed to pellet cells and cell-free supernatants isolated. The Nano-GloⓇ HiBiT Extracellular Detection System was used according to the manufacturer’s instructions (Promega). Relative light units (RLU) were measured using a BioTek Synergy plate reader using an integration time of 2 s. RLUs were converted to concentrations by extrapolating to a standard curve generated using the HiBiT Control Protein (Promega).

### Peptide cleavage prediction scores

*ECE1* P2-P3 amino acid sequences were parsed using ProP 1.0 Server (http://www.cbs.dtu.dk/services/ProP/), a tool based on the furin protease (a mammalian homolog of Kex2p) to predict proteins cleaved at KR residues and generate cleavage indices [21].

### *ECE1* sequence genotyping and analysis

*ECE1* sequences from a collection of deidentified *C*. *albicans* vaginal isolates (n = 12, obtained from Detroit Vaginitis Clinic, Detroi, MI, USA) and various other isolates (n = 66, obtained from First Affiliated Hospital of Guangzhou Medical University, First Affiliated Hospital of Jinan University, Third Affiliated Hospital Sun Yat-sen University, Zhujiang Hospital of Southern Medical University, and Guangdong Provincial Hospital of Chinese Medicine in Guangzhou, China) were obtained by extracting genomic DNA as described previously [47]. The P2-P3 sequences were PCR amplified using high fidelity polymerase and degenerate primers P2P3invarSEQ-F and P2P3invarSEQ-R, purified by spin column and sequence established by Sanger methodology. Trace files were analyzed to confirm sequence integrity. P2-P3 sequences from clinical isolates were translated using the alternative yeast nuclear codon table, aligned with ClustalW, and parsed using WebLogo (https://weblogo.berkeley.edu/) to determine consensus amino acid sequences [48,49].

### Statistics

All datasets were tested for normality using the Shapiro-Wilk test. Datasets that were normally distributed were tested for significance using either two-tailed Student’s t-test or one-way ANOVA with Dunnet’s post-test. Non-parametric datasets were assessed using Mann-Whitney U test or Kruskal-Wallis with Dunn’s post-test. Linear regression analyses were conducted by calculating r^2^ values to determine goodness of fit. Results were considered statistically significant at p < 0.05. All statistical tests were conducted using GraphPad Prism 8.

## Supporting information

S1 Fig Differential promoter activity does not explain reduced pathogenicity of the variant *ECE1* allele. Isogenic strains were constructed to express either WT (TWO) or VAR (TVO) *ECE1* alleles from the constitutive *TEF1* promoter. **(A)** *In vitro ECE1* expression levels were measured by qRT-PCR 4 h after strains were transferred to RPMI-1640. Data were normalized to *ACT1* expression and TWO using the ΔΔCt method (mean ± SD). Mice (n = 8) were challenged with TWO or TVO strains and vaginal lavage performed at d 3 post-infection. Lavage fluids were assessed for **(B)** CFU by microbiological plating (median ± IQR), **(C)** PMN recruitment by microscopy (median ± IQR), **(D)** IL-1β by ELISA (median ± IQR), and **(E)** tissue damage by LDH assay (median ± IQR). Statistical significance was evaluated by Mann-Whitney U test. All in vitro experiments were conducted in biological triplicate. *, p < 0.05, ** p < 0.01.
(TIF)

S2 Fig Validation of strains expressing chimeric *ECE1* alleles. **(A and B)***In vitro ECE1* expression levels were measured by qRT-PCR 4 h after strains were transferred to RPMI-1640 and normalized to both *ACT1* expression and WO values using the ΔΔCt method (mean ± SD). Statistical significance was assessed using one-way ANOVA and Dunnet’s post-test. **(C)** Strains were grown in YPD medium and OD600 values measured by spectrophotometer (mean ± SD). **(D)** Strains were adjusted to 1x10^6^ cells/mL in RPMI-1640 and imaged by light microscopy at 4 h to assess hyphal growth. Figure depicts representative images from three independent experiments.
(TIF)

S3 Fig Candidalysin secretion is correlated with IL-1β release and damage. HiBiT-tagged candidalysin secretion (Fig 6H) was plotted against **(A)** IL-1β release (Fig 6F) and **(B)** LDH levels (Fig 6G). Linear regression analysis was conducted and r^2^ values calculated.
(TIF)

S1 Table Strains used or constructed in this study. (DOCX)

S2 Table Primers used for strain construction, quantitative PCR, or *ECE1* locus genotyping. (DOCX)

S3 Table Sequences of gBlocks used to construct *ECE1* ORF and peptide swap strains. (DOCX)

S4 Table *ECE1* P2 and candidalysin sequences from *C*. *albicans* clinical isolates. (DOCX)
