# Supplementary figures and images for: A variant ECE1 allele contributes to reduced pathogenicity of Candida albicans during vulvovaginal candidiasis
Source: PLoS Pathog. 2021 Sep 10;17(9):e1009884. doi: 10.1371/journal.ppat.1009884 (PMC8432879; doi:10.1371/journal.ppat.1009884)

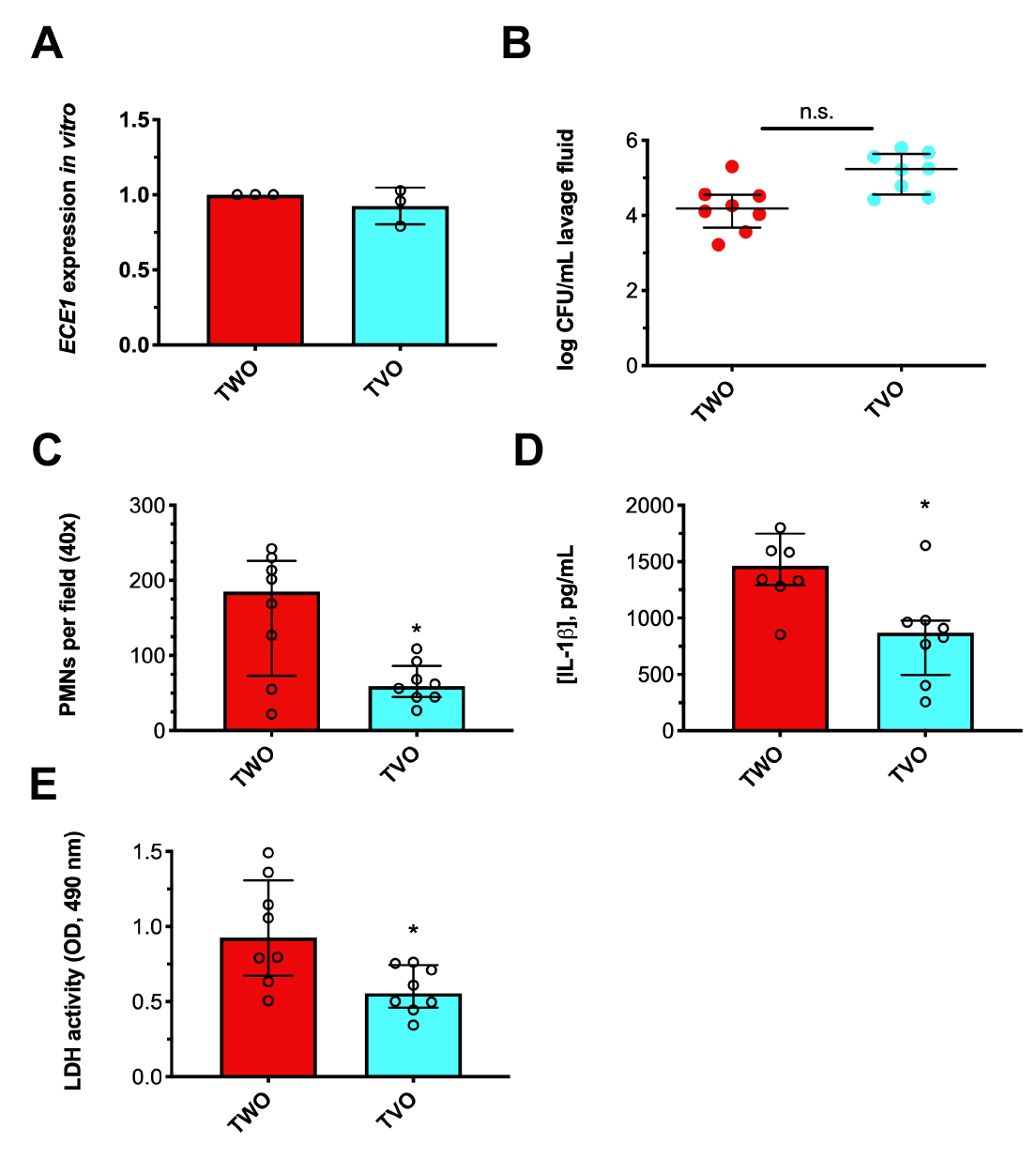

Supplement: S1 Fig — Isogenic strains were constructed to express either WT (TWO) or VAR (TVO) ECE1 alleles from the constitutive TEF1 promoter. (A) In vitro ECE1 expression levels were measured by qRT-PCR 4 h after strains were transferred to RPMI-1640. Data were normalized to ACT1 expression and TWO using the ΔΔCt method (mean ± SD). Mice (n = 8) were challenged with TWO or TVO strains and vaginal lavage performed at d 3 post-infection. Lavage fluids were assessed for (B) CFU by microbiological plating (median ± IQR), (C) PMN recruitment by microscopy (median ± IQR), (D) IL-1β by ELISA (median ± IQR), and (E) tissue damage by LDH assay (median ± IQR). Statistical significance was evaluated by Mann-Whitney U test. All in vitro experiments were conducted in biological triplicate. *, p < 0.05, ** p < 0.01. (TIF) [file ppat.1009884.s001.tif]

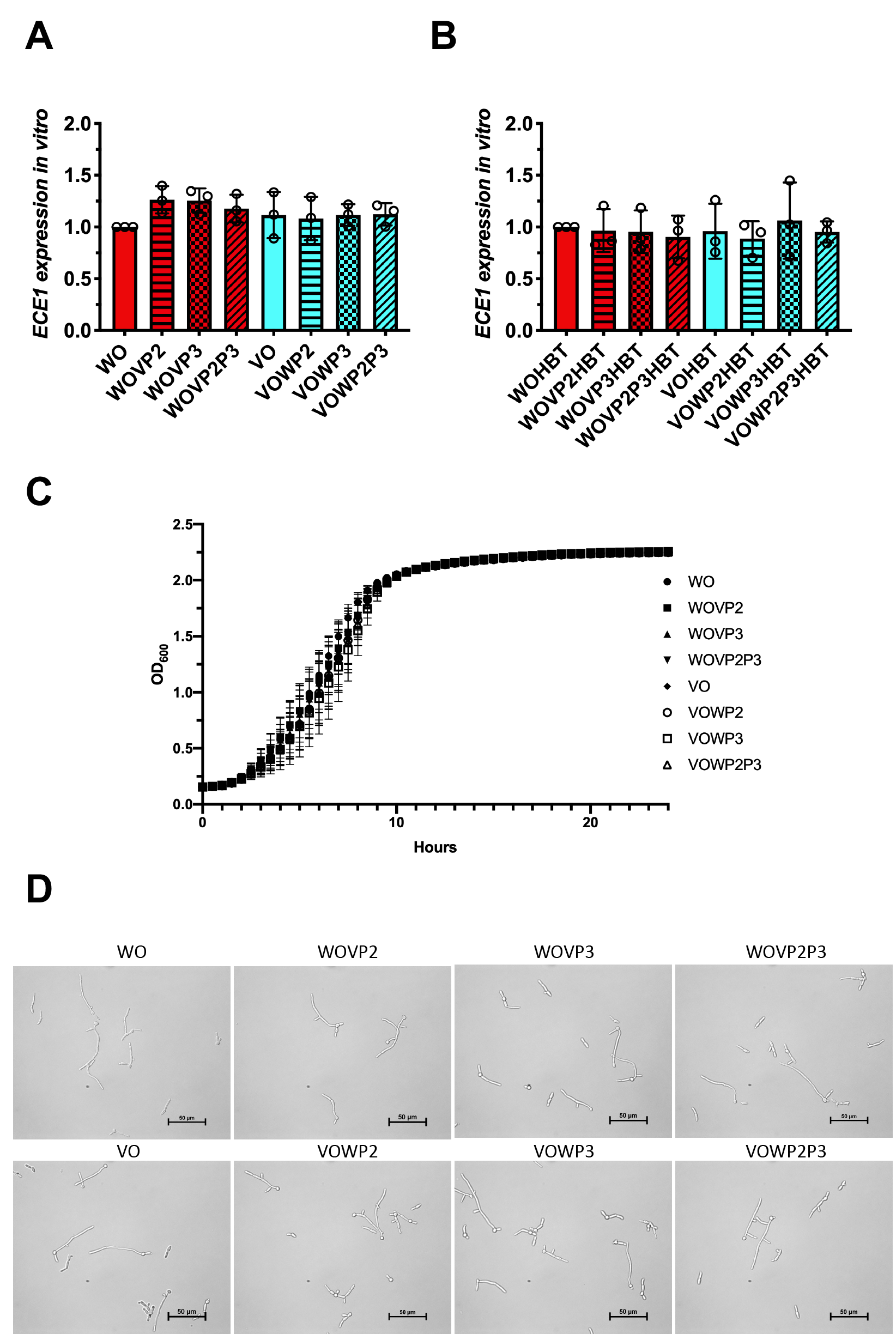

Supplement: S2 Fig — (A and B)In vitro ECE1 expression levels were measured by qRT-PCR 4 h after strains were transferred to RPMI-1640 and normalized to both ACT1 expression and WO values using the ΔΔCt method (mean ± SD). Statistical significance was assessed using one-way ANOVA and Dunnet’s post-test. (C) Strains were grown in YPD medium and OD600 values measured by spectrophotometer (mean ± SD). (D) Strains were adjusted to 1x10^6^ cells/mL in RPMI-1640 and imaged by light microscopy at 4 h to assess hyphal growth. Figure depicts representative images from three independent experiments. (TIF) [file ppat.1009884.s002.tif]

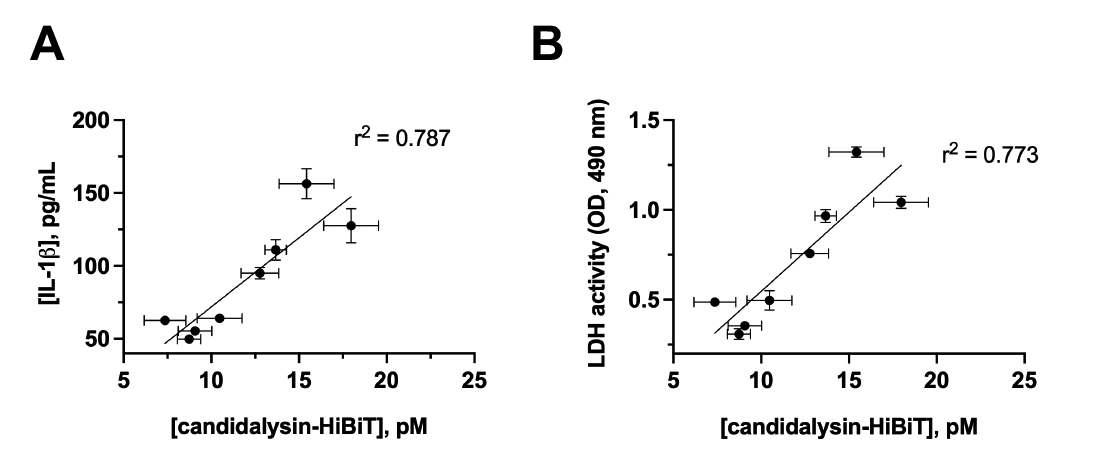

Supplement: S3 Fig — HiBiT-tagged candidalysin secretion (Fig 6H) was plotted against (A) IL-1β release (Fig 6F) and (B) LDH levels (Fig 6G). Linear regression analysis was conducted and r2 values calculated. (TIF) [file ppat.1009884.s003.tif]
