# Supplementary material for: A variant ECE1 allele contributes to reduced pathogenicity of Candida albicans during vulvovaginal candidiasis
Source: PLoS Pathog. 2021 Sep 10;17(9):e1009884. doi: 10.1371/journal.ppat.1009884 (PMC8432879; doi:10.1371/journal.ppat.1009884)
Supplement: S1 Table — (DOCX) [file ppat.1009884.s004.docx]

**S1 Table. Strains used or constructed in this study.**

| **Strain** | **Parent** | **Genotype** | **Reference** |
| --- | --- | --- | --- |
| SC5314 | n/a | wild-type reference strain | [1] |
| 529L | n/a | clinical isolate | [2] |
| BWP17 | CAI4 | *ura3*Δ/Δ *his1*Δ/Δ *arg4*Δ/Δ | [3] |
| *ece1*Δ/Δ *ura3*Δ/Δ | BWP17 | *ura3*Δ/Δ *ece1Δ*::*HIS1* *ece1Δ*::*ARG4* | This study |
| GP1 | BWP17 | *ura3*Δ::*URA3 his1*Δ::*HIS1* *arg4*Δ::*ARG4* | [4] |
| 3kbWO | *ece1*Δ/Δ *ura3*Δ/Δ | *ece1Δ*::*HIS1* *ece1Δ*::*ARG4 ura3*Δ::*URA3*-PrECE1_SC5314_-*ECE1*_SC5314-_tECE1 | This study |
| 3kbVO | *ece1*Δ/Δ *ura3*Δ/Δ | *ece1Δ*::*HIS1* *ece1Δ*::*ARG4 ura3*Δ::*URA3*-PrECE1_529L_-*ECE1*_529L-_tECE1 | This study |
| EV | *ece1*Δ/Δ *ura3*Δ/Δ | *ece1Δ*::*HIS1* *ece1Δ*::*ARG4 ura3*Δ::*URA3* | This study |
| TWO | *ece1*Δ/Δ *ura3*Δ/Δ | *ece1Δ*::*HIS1* *ece1Δ*::*ARG4 ura3*Δ::*URA3*-PrTEF1-*ECE1*_SC5314_-tADH1 | This study |
| TVO | *ece1*Δ/Δ *ura3*Δ/Δ | *ece1Δ*::*HIS1* *ece1Δ*::*ARG4 ura3*Δ::*URA3*-PrTEF1-*ECE1*_529L_-tADH1 | This study |
| WO | *ece1*Δ/Δ *ura3*Δ/Δ | *ece1Δ*::*HIS1* *ece1Δ*::*ARG4 ura3*Δ::*URA3*-PrECE1_SC5314_-*ECE1*_SC5314_-tADH1 | This study |
| WOVP2 | *ece1*Δ/Δ *ura3*Δ/Δ | *ece1Δ*::*HIS1* *ece1Δ*::*ARG4 ura3*Δ::*URA3*-PrECE1_SC5314_-*ECE1*_SC5314_P2_529L_-tADH1 | This study |
| WOVP3 | *ece1*Δ/Δ *ura3*Δ/Δ | *ece1Δ*::*HIS1* *ece1Δ*::*ARG4 ura3*Δ::*URA3*-PrECE1_SC5314_-*ECE1*_SC5314_P3_529L_-tADH1 | This study |
| WOVP2P3 | *ece1*Δ/Δ *ura3*Δ/Δ | *ece1Δ*::*HIS1* *ece1Δ*::*ARG4 ura3*Δ::*URA3*-PrECE1_SC5314_-*ECE1*_SC5314_P2P3_529L_-tADH1 | This study |
| VO | *ece1*Δ/Δ *ura3*Δ/Δ | *ece1Δ*::*HIS1* *ece1Δ*::*ARG4 ura3*Δ::*URA3*-PrECE1_SC5314_-*ECE1*_529L_-tADH1 | This study |
| VOWP2 | *ece1*Δ/Δ *ura3*Δ/Δ | *ece1Δ*::*HIS1* *ece1Δ*::*ARG4 ura3*Δ::*URA3*-PrECE1_SC5314_-*ECE1*_529L_P2_SC5314_-tADH1 | This study |
| VOWP3 | *ece1*Δ/Δ *ura3*Δ/Δ | *ece1Δ*::*HIS1* *ece1Δ*::*ARG4 ura3*Δ::*URA3*-PrECE1_SC5314_-*ECE1*_529L_P3_SC5314_-tADH1 | This study |
| VOWP2P3 | *ece1*Δ/Δ *ura3*Δ/Δ | *ece1Δ*::*HIS1* *ece1Δ*::*ARG4 ura3*Δ::*URA3*-PrECE1_SC5314_-*ECE1*_529L_P2P3_SC5314_-tADH1 | This study |
| WOHBT | *ece1*Δ/Δ *ura3*Δ/Δ | *ece1Δ*::*HIS1* *ece1Δ*::*ARG4 ura3*Δ::*URA3*-PrECE1_SC5314_-*ECE1*_SC5314_-clysHiBiT-tADH1 | This study |
| WOVP2HBT | *ece1*Δ/Δ *ura3*Δ/Δ | *ece1Δ*::*HIS1* *ece1Δ*::*ARG4 ura3*Δ::*URA3*-PrECE1_SC5314_-*ECE1*_SC5314_P2_529L_-clysHiBiT-tADH1 | This study |
| WOVP3HBT | *ece1*Δ/Δ *ura3*Δ/Δ | *ece1Δ*::*HIS1* *ece1Δ*::*ARG4 ura3*Δ::*URA3*-PrECE1_SC5314_-*ECE1*_SC5314_P3_529L_-clysHiBiT-tADH1 | This study |
| WOVP2P3HBT | *ece1*Δ/Δ *ura3*Δ/Δ | *ece1Δ*::*HIS1* *ece1Δ*::*ARG4 ura3*Δ::*URA3*-PrECE1_SC5314_-*ECE1*_SC5314_P2P3_529L_-clysHiBiT-tADH1 | This study |
| VOHBT | *ece1*Δ/Δ *ura3*Δ/Δ | *ece1Δ*::*HIS1* *ece1Δ*::*ARG4 ura3*Δ::*URA3*-PrECE1_SC5314_-*ECE1*_529L_-clysHiBiT-tADH1 | This study |
| VOWP2HBT | *ece1*Δ/Δ *ura3*Δ/Δ | *ece1Δ*::*HIS1* *ece1Δ*::*ARG4 ura3*Δ::*URA3*-PrECE1_SC5314_-*ECE1*_529L_P2_SC5314_-clysHiBiT-tADH1 | This study |
| VOWP3HBT | *ece1*Δ/Δ *ura3*Δ/Δ | *ece1Δ*::*HIS1* *ece1Δ*::*ARG4 ura3*Δ::*URA3*-PrECE1_SC5314_-*ECE1*_529L_P3_SC5314_-clysHiBiT-tADH1 | This study |
| VOWP2P3HBT | *ece1*Δ/Δ *ura3*Δ/Δ | *ece1Δ*::*HIS1* *ece1Δ*::*ARG4 ura3*Δ::*URA3*-PrECE1_SC5314_-*ECE1*_529L_P2P3_SC5314_-clysHiBiT-tADH1 | This study |
| 529L-WO | 529L | *ECE1*/*ECE1* Δ*neut5L*::*NAT1*-PrECE1_SC5314_-*ECE1*_SC5314_-tADH1/*NEUT5L* | This study |
| 529L-VO | 529L | *ECE1*/*ECE1* Δ*neut5L*::*NAT1*-PrECE1_SC5314_-*ECE1*_529L_-tADH1/*NEUT5L* | This study |
| SC5314-WOHBT | SC5314 | *ECE1*/*ECE1* Δ*neut5L*::*NAT1*-PrECE1_SC5314_-*ECE1*_SC5314_-clys-HiBiT-tADH1/*NEUT5L* | This study |
| SC5314-VOHBT | SC5314 | *ECE1*/*ECE1* Δ*neut5L*::*NAT1*-PrECE1_SC5314_-*ECE1*_529L_-clys-HiBiT-tADH1/*NEUT5L* | This study |
| 529L-WOHBT | 529L | *ECE1*/*ECE1* Δ*neut5L*::*NAT1*-PrECE1_SC5314_-*ECE1*_SC5314_-clys-HiBiT-tADH1/*NEUT5L* | This study |
| 529L-VOHBT | 529L | *ECE1*/*ECE1* Δ*neut5L*::*NAT1*-PrECE1_SC5314_-*ECE1*_529L_-clys-HiBiT-tADH1/*NEUT5L* | This study |

**References**

1. Gillum AM, Tsay EY, Kirsch DR (1984) Isolation of the *Candida albicans* gene for orotidine-5'-phosphate decarboxylase by complementation of *S. cerevisiae ura3* and *E. coli* pyrF mutations. Mol Gen Genet 198: 179-182.

2. Rahman D, Mistry M, Thavaraj S, Challacombe SJ, Naglik JR (2007) Murine model of concurrent oral and vaginal *Candida albicans* colonization to study epithelial host-pathogen interactions. Microbes Infect 9: 615-622.

3. Wilson RB, Davis D, Mitchell AP (1999) Rapid hypothesis testing with *Candida albicans* through gene disruption with short homology regions. J Bacteriol 181: 1868-1874.

4. Willems HME, Bruner WS, Barker KS, Liu J, Palmer GE, et al. (2017) Overexpression of *Candida albicans* Secreted Aspartyl Proteinases 2 or 5 is not sufficient for exacerbation of immunopathology in a murine model of vaginitis. Infect Immun 85: e00248-17.
