# Supplementary material for: A variant ECE1 allele contributes to reduced pathogenicity of Candida albicans during vulvovaginal candidiasis
Source: PLoS Pathog. 2021 Sep 10;17(9):e1009884. doi: 10.1371/journal.ppat.1009884 (PMC8432879; doi:10.1371/journal.ppat.1009884)
Supplement: S2 Table — (DOCX) [file ppat.1009884.s005.docx]

**S2 Table. Primers used for strain construction, quantitative PCR, or *ECE1* locus genotyping.**

| **Function** | **Name** | **Sequence** |
| --- | --- | --- |
| *ECE1* deletion and confirmation | ECE1DISF | GTTTATCTCTACAACAAACAACTTTCCTTTATTTTACTACCAACTATTTTCCATTCGTTAAAGTTTTCCCAGTCACGACGTT |
|  | ECE1DISR | GTGTCACAAGACTTATGGAATAAAAGATTAAGCTTGTGGAAAACAAATTTTTATCTGCTGAGCATTGTGGAATTGTGAGCGGATA |
|  | ECE1AMPF | GGATCCCGATACATTGTAGTC |
|  | ECE1AMPR | CCAGACGTTGGTTGCAAAAC |
|  | HISINTF2 | ACTGTATCCTCTTCTGTCCCC |
|  | HISINTR2 | CGACCATATGGGAGAGCTCCC |
|  | ARGINTF2 | AAGCTAGTGTGGAAAGAAGAG |
|  | ARGINTR2 | AATGACTGAATTATGTCGGTC |
|  | ECE1DETF | GGCAACATTCCACAAGTAATC |
|  | ECE1DETR | GCTGATCTAGTAATGAGTTGTGG |
|  | LUXINTDETF | CTGACCTTTAGTCTTTCCTGC |
|  | LUXINTDETR | CAGTAGTACTTGTTGTTGTATCG |
| Native allele strains | ECE1Pr3k-F-StuI | TCA***AGGCCT***TCCAGTTGCCCTACTTACTGATATACATTC |
|  | tECE1-R-SacI | TCA***GAGCTC***GATTTACCCCAGACGTTGGTTGC |
| Overexpression strains | ECE1ORFF-SalI (PAGE) | TCA***GTCGAC***ATGAAATTCTCCAAAATTGCCTGTGC |
|  | ECE1ORFR-MluI | TCA***ACGCGT***ACAAATTTTTATCTGCTGAGC |
| GFPy reporter | PrECE1-R-SalI | TCA***GTCGAC***TTTAACGAATGGAAAATAGTTGGTAG |
| SynthGene primers | SynthGene-F | CGCAGTTACGGATCAGTCAC |
|  | SynthGene-Rv3 | AGCAAAGAACATGGCAGCACC |
| qPCR primers | ECE1invarQPCR-F | GAAGATATTGATTCTGTTGTTGCTGG |
|  | ECE1invarQPCR-R | CAGTTTCCAGGACGCCAT |
|  | ACT1invarQPCR-F | CCCAATTGAACGCGGTATTGTTTC |
|  | ACT1invarQPCR-R | CAACGTGAGTAACGCCATCACC |
| pDUP3 Cloning | PrECE1-F-ClaI | ATCA***ATCGAT***GACGGCCAGTCCGTAATACGA |
|  | ADHtermv2-R-SpeI | TCA***ACTAGT***CCGAAAACTTGAAACTTGAAAACACCGAGTTG |
|  | NEUT5LAMPR | Ggaatttctagtcacttgacacgacc |
|  | NAT1INTF | CCCAGATGCGAAGTTAAGTGCG |
|  | NEUT5LAMPF | gctgaatcacttgataggatttagttccattatgg |
| Sequencing primers | ECE1ProSEQF4 | GCCCGCCCACAAATCTTAC |
|  | pLUXURA3SEQR | CTGGCCAATTATAAATGTGAAGG |
|  | TEF1PRSEQF | TTTTTGCTGTTCACTTTCTCG |
|  | ADH13SEQR | ATATCGCACTCACGTAAACAC |
|  | P2P3invarSeq-F | CCATGCTCCAGAATTCAACATGAARAGA |
|  | P2P3invarSeq-R | CAGTTTCCAGGACGCCAT |

Underlined sequence indicate complementarity to plasmids pGEMHIS1 and pRSARG4ΔSpe. Bold, italic sequences denote restriction sites.
