# Supplementary material for: A variant ECE1 allele contributes to reduced pathogenicity of Candida albicans during vulvovaginal candidiasis
Source: PLoS Pathog. 2021 Sep 10;17(9):e1009884. doi: 10.1371/journal.ppat.1009884 (PMC8432879; doi:10.1371/journal.ppat.1009884)
Supplement: S3 Table — (DOCX) [file ppat.1009884.s006.docx]

**S3 Table. Sequences of gBlocks used to construct *ECE1* ORF and peptide swap strains.**

| **Name** | **Sequence** |
| --- | --- |
| WO | CGCAGTTACGGATCAGTCACTCAGTCGACATGAAATTCTCCAAAATTGCCTGTGCTACTGTTTTTGCTTTATCTTCTCAAGCTGCCATCATCCACCATGCTCCAGAATTCAACATGAAGAGAGATGTTGCTCCAGCTGCCCCAGCTGCTCCAGCTGACCAAGCACCTACTGTTCCTGCACCTCAAGAATTCAATACTGCTATTACCAAAAGAAGTATTATTGGAATTATTATGGGTATTCTTGGCAACATTCCACAAGTAATCCAAATCATCATGAGTATTGTCAAAGCTTTCAAAGGTAACAAGAGAGAAGATATTGATTCTGTTGTTGCTGGTATCATTGCTGATATGCCATTTGTTGTCAGAGCTGTTGACACAGCCATGACTTCTGTTGCTTCTACCAAGAGAGATGGAGCTAATGATGACGTTGCTAATGCCGTCGTCAGATTGCCAGAAATTGTTGCTCGTGTTGCCACTGGTGTTCAACAATCCATCGAAAATGCCAAGAGAGATGGCGTTCCAGATGTTGGCCTTAATCTTGTTGCTAATGCTCCAAGACTTATCTCTAACGTTTTTGATGGCGTCCTGGAAACTGTTCAACAAGCTAAGAGAGATGGTCTTGAAGATTTTCTTGATGAACTTCTTCAAAGACTCCCACAACTCATTACTAGATCAGCTGAATCTGCTTTGAAAGACAGTCAACCAGTTAAAAGAGATGCCGGCTCAGTAGCACTTAGCAATTTAATCAAAAAGAGCATTGAAACTGTCGGTATTGAAAATGCTGCTCAAATTGTTTCAGAAAGAGATATTTCTTCTTTGATTGAAGAATATTTCGGAAAAGCTTAAACGCGTTCAGGTGCTGCCATGTTCTTTGCT |
| WOVP2 | CGCAGTTACGGATCAGTCACTCAGTCGACATGAAATTCTCCAAAATTGCCTGTGCTACTGTTTTTGCTTTATCTTCTCAAGCTGCCATCATCCACCATGCTCCAGAATTCAACATGAAGAGAGATGTTGTCCCAGCTGGTCAAGGTGATCCAGCCTCTGGCCCAGAACCTCAACTTGCTCCTGCACCTCAAGGAATCAACACTGATCTTGCCAAAAGAAGTATTATTGGAATTATTATGGGTATTCTTGGCAACATTCCACAAGTAATCCAAATCATCATGAGTATTGTCAAAGCTTTCAAAGGTAACAAGAGAGAAGATATTGATTCTGTTGTTGCTGGTATCATTGCTGATATGCCATTTGTTGTCAGAGCTGTTGACACAGCCATGACTTCTGTTGCTTCTACCAAGAGAGATGGAGCTAATGATGACGTTGCTAATGCCGTCGTCAGATTGCCAGAAATTGTTGCTCGTGTTGCCACTGGTGTTCAACAATCCATCGAAAATGCCAAGAGAGATGGCGTTCCAGATGTTGGCCTTAATCTTGTTGCTAATGCTCCAAGACTTATCTCTAACGTTTTTGATGGCGTCCTGGAAACTGTTCAACAAGCTAAGAGAGATGGTCTTGAAGATTTTCTTGATGAACTTCTTCAAAGACTCCCACAACTCATTACTAGATCAGCTGAATCTGCTTTGAAAGACAGTCAACCAGTTAAAAGAGATGCCGGCTCAGTAGCACTTAGCAATTTAATCAAAAAGAGCATTGAAACTGTCGGTATTGAAAATGCTGCTCAAATTGTTTCAGAAAGAGATATTTCTTCTTTGATTGAAGAATATTTCGGAAAAGCTTAAACGCGTTCAGGTGCTGCCATGTTCTTTGCT |
| WOVP3 | CGCAGTTACGGATCAGTCACTCAGTCGACATGAAATTCTCCAAAATTGCCTGTGCTACTGTTTTTGCTTTATCTTCTCAAGCTGCCATCATCCACCATGCTCCAGAATTCAACATGAAGAGAGATGTTGCTCCAGCTGCCCCAGCTGCTCCAGCTGACCAAGCACCTACTGTTCCTGCACCTCAAGAATTCAATACTGCTATTACCAAAAGAAGTTTTCTTAGTATTATCACTGCTCTTCTTGGAAACATTCCACAAATAATCCAAATCATCATGGGCATTGTCAAAGCTTTCAGAGGTAACAAGAGAGAAGATATTGATTCTGTTGTTGCTGGTATCATTGCTGATATGCCATTTGTTGTCAGAGCTGTTGACACAGCCATGACTTCTGTTGCTTCTACCAAGAGAGATGGAGCTAATGATGACGTTGCTAATGCCGTCGTCAGATTGCCAGAAATTGTTGCTCGTGTTGCCACTGGTGTTCAACAATCCATCGAAAATGCCAAGAGAGATGGCGTTCCAGATGTTGGCCTTAATCTTGTTGCTAATGCTCCAAGACTTATCTCTAACGTTTTTGATGGCGTCCTGGAAACTGTTCAACAAGCTAAGAGAGATGGTCTTGAAGATTTTCTTGATGAACTTCTTCAAAGACTCCCACAACTCATTACTAGATCAGCTGAATCTGCTTTGAAAGACAGTCAACCAGTTAAAAGAGATGCCGGCTCAGTAGCACTTAGCAATTTAATCAAAAAGAGCATTGAAACTGTCGGTATTGAAAATGCTGCTCAAATTGTTTCAGAAAGAGATATTTCTTCTTTGATTGAAGAATATTTCGGAAAAGCTTAAACGCGTTCAGGTGCTGCCATGTTCTTTGCT |
| WOVP2P3 | CGCAGTTACGGATCAGTCACTCAGTCGACATGAAATTCTCCAAAATTGCCTGTGCTACTGTTTTTGCTTTATCTTCTCAAGCTGCCATCATCCACCATGCTCCAGAATTCAACATGAAGAGAGATGTTGTCCCAGCTGGTCAAGGTGATCCAGCCTCTGGCCCAGAACCTCAACTTGCTCCTGCACCTCAAGGAATCAACACTGATCTTGCCAAAAGAAGTTTTCTTAGTATTATCACTGCTCTTCTTGGAAACATTCCACAAATAATCCAAATCATCATGGGCATTGTCAAAGCTTTCAGAGGTAACAAGAGAGAAGATATTGATTCTGTTGTTGCTGGTATCATTGCTGATATGCCATTTGTTGTCAGAGCTGTTGACACAGCCATGACTTCTGTTGCTTCTACCAAGAGAGATGGAGCTAATGATGACGTTGCTAATGCCGTCGTCAGATTGCCAGAAATTGTTGCTCGTGTTGCCACTGGTGTTCAACAATCCATCGAAAATGCCAAGAGAGATGGCGTTCCAGATGTTGGCCTTAATCTTGTTGCTAATGCTCCAAGACTTATCTCTAACGTTTTTGATGGCGTCCTGGAAACTGTTCAACAAGCTAAGAGAGATGGTCTTGAAGATTTTCTTGATGAACTTCTTCAAAGACTCCCACAACTCATTACTAGATCAGCTGAATCTGCTTTGAAAGACAGTCAACCAGTTAAAAGAGATGCCGGCTCAGTAGCACTTAGCAATTTAATCAAAAAGAGCATTGAAACTGTCGGTATTGAAAATGCTGCTCAAATTGTTTCAGAAAGAGATATTTCTTCTTTGATTGAAGAATATTTCGGAAAAGCTTAAACGCGTTCAGGTGCTGCCATGTTCTTTGCT |
| VO | CGCAGTTACGGATCAGTCACTCAGTCGACATGAAATTCTCCAAAATTGCCTGTGCTACTGTTTTTGCTTTATCTTCTCAAGCTGCCATCATTCACCATGCTCCAGAATTCAACATGAAGAGAGATGTTGTCCCAGCTGGTCAAGGTGATCCAGCCTCTGGCCCAGAACCTCAACTTGCTCCTGCACCTCAAGGAATCAACACTGATCTTGCCAAAAGAAGTTTTCTTAGTATTATCACTGCTCTTCTTGGAAACATTCCACAAATAATCCAAATCATCATGGGCATTGTCAAAGCTTTCAGAGGTAACAAGAGAGAAGATATTGATTCTGTTGTTGCTGGTATCATTGCTGATATGCCATTTGTTGCTAGAGCTGTTGACACAGCCATGACTTCTGTTGCTTCCACCAAGAGAGATGGAGCTAACGATGACGTTGCTAATGCCGTCGTCAGATTGCCAGAAATTGTTGCTCGTGTTGCCACTGGTGTTCAACAATCCATCGAAAATGCCAAGAGAGATGGCGTTCCAGACGTTGGTCTTAATCTTGTTGCTAATGCCCCAAGACTTATCTCTGACGTTTTTGATGGCGTCCTGGAAACTGTTCAACAAGCTAAGAGAGATGGTCTTGAAGATGCTCTTAATGAACTTCTTGAGCAACTCCCAAAACTTATTACTAGATCGGCTGAATCTGCTTTGAAAGACAGTCAACCAGTTAAAAGAGATGCCGGTTCAGTAGCACTTAGCAATTTAATTAAGAAAAGTATTGAAACTGTCGGTATTGAAAATGCTGCTCAAATTGTTTCAGAAAGAGATATTTCTTCTTTGATTGAAGAATATTTCGGAAATGCTTAAACGCGTTCAGGTGCTGCCATGTTCTTTGCT |
| VOWP2 | CGCAGTTACGGATCAGTCACTCAGTCGACATGAAATTCTCCAAAATTGCCTGTGCTACTGTTTTTGCTTTATCTTCTCAAGCTGCCATCATTCACCATGCTCCAGAATTCAACATGAAGAGAGATGTTGCTCCAGCTGCCCCAGCTGCTCCAGCTGACCAAGCACCTACTGTTCCTGCACCTCAAGAATTCAATACTGCTATTACCAAAAGAAGTTTTCTTAGTATTATCACTGCTCTTCTTGGAAACATTCCACAAATAATCCAAATCATCATGGGCATTGTCAAAGCTTTCAGAGGTAACAAGAGAGAAGATATTGATTCTGTTGTTGCTGGTATCATTGCTGATATGCCATTTGTTGCTAGAGCTGTTGACACAGCCATGACTTCTGTTGCTTCCACCAAGAGAGATGGAGCTAACGATGACGTTGCTAATGCCGTCGTCAGATTGCCAGAAATTGTTGCTCGTGTTGCCACTGGTGTTCAACAATCCATCGAAAATGCCAAGAGAGATGGCGTTCCAGACGTTGGTCTTAATCTTGTTGCTAATGCCCCAAGACTTATCTCTGACGTTTTTGATGGCGTCCTGGAAACTGTTCAACAAGCTAAGAGAGATGGTCTTGAAGATGCTCTTAATGAACTTCTTGAGCAACTCCCAAAACTTATTACTAGATCGGCTGAATCTGCTTTGAAAGACAGTCAACCAGTTAAAAGAGATGCCGGTTCAGTAGCACTTAGCAATTTAATTAAGAAAAGTATTGAAACTGTCGGTATTGAAAATGCTGCTCAAATTGTTTCAGAAAGAGATATTTCTTCTTTGATTGAAGAATATTTCGGAAATGCTTAAACGCGTTCAGGTGCTGCCATGTTCTTTGCT |
| VOWP3 | CGCAGTTACGGATCAGTCACTCAGTCGACATGAAATTCTCCAAAATTGCCTGTGCTACTGTTTTTGCTTTATCTTCTCAAGCTGCCATCATTCACCATGCTCCAGAATTCAACATGAAGAGAGATGTTGTCCCAGCTGGTCAAGGTGATCCAGCCTCTGGCCCAGAACCTCAACTTGCTCCTGCACCTCAAGGAATCAACACTGATCTTGCCAAAAGAAGTATTATTGGAATTATTATGGGTATTCTTGGCAACATTCCACAAGTAATCCAAATCATCATGAGTATTGTCAAAGCTTTCAAAGGTAACAAGAGAGAAGATATTGATTCTGTTGTTGCTGGTATCATTGCTGATATGCCATTTGTTGCTAGAGCTGTTGACACAGCCATGACTTCTGTTGCTTCCACCAAGAGAGATGGAGCTAACGATGACGTTGCTAATGCCGTCGTCAGATTGCCAGAAATTGTTGCTCGTGTTGCCACTGGTGTTCAACAATCCATCGAAAATGCCAAGAGAGATGGCGTTCCAGACGTTGGTCTTAATCTTGTTGCTAATGCCCCAAGACTTATCTCTGACGTTTTTGATGGCGTCCTGGAAACTGTTCAACAAGCTAAGAGAGATGGTCTTGAAGATGCTCTTAATGAACTTCTTGAGCAACTCCCAAAACTTATTACTAGATCGGCTGAATCTGCTTTGAAAGACAGTCAACCAGTTAAAAGAGATGCCGGTTCAGTAGCACTTAGCAATTTAATTAAGAAAAGTATTGAAACTGTCGGTATTGAAAATGCTGCTCAAATTGTTTCAGAAAGAGATATTTCTTCTTTGATTGAAGAATATTTCGGAAATGCTTAAACGCGTTCAGGTGCTGCCATGTTCTTTGCT |
| VOWP2P3 | CGCAGTTACGGATCAGTCACTCAGTCGACATGAAATTCTCCAAAATTGCCTGTGCTACTGTTTTTGCTTTATCTTCTCAAGCTGCCATCATTCACCATGCTCCAGAATTCAACATGAAGAGAGATGTTGCTCCAGCTGCCCCAGCTGCTCCAGCTGACCAAGCACCTACTGTTCCTGCACCTCAAGAATTCAATACTGCTATTACCAAAAGAAGTATTATTGGAATTATTATGGGTATTCTTGGCAACATTCCACAAGTAATCCAAATCATCATGAGTATTGTCAAAGCTTTCAAAGGTAACAAGAGAGAAGATATTGATTCTGTTGTTGCTGGTATCATTGCTGATATGCCATTTGTTGCTAGAGCTGTTGACACAGCCATGACTTCTGTTGCTTCCACCAAGAGAGATGGAGCTAACGATGACGTTGCTAATGCCGTCGTCAGATTGCCAGAAATTGTTGCTCGTGTTGCCACTGGTGTTCAACAATCCATCGAAAATGCCAAGAGAGATGGCGTTCCAGACGTTGGTCTTAATCTTGTTGCTAATGCCCCAAGACTTATCTCTGACGTTTTTGATGGCGTCCTGGAAACTGTTCAACAAGCTAAGAGAGATGGTCTTGAAGATGCTCTTAATGAACTTCTTGAGCAACTCCCAAAACTTATTACTAGATCGGCTGAATCTGCTTTGAAAGACAGTCAACCAGTTAAAAGAGATGCCGGTTCAGTAGCACTTAGCAATTTAATTAAGAAAAGTATTGAAACTGTCGGTATTGAAAATGCTGCTCAAATTGTTTCAGAAAGAGATATTTCTTCTTTGATTGAAGAATATTTCGGAAATGCTTAAACGCGTTCAGGTGCTGCCATGTTCTTTGCT |
| WOHBT | CGCAGTTACGGATCAGTCACTCAGTCGACATGAAATTCTCCAAAATTGCCTGTGCTACTGTTTTTGCTTTATCTTCTCAAGCTGCCATCATCCACCATGCTCCAGAATTCAACATGAAGAGAGATGTTGCTCCAGCTGCCCCAGCTGCTCCAGCTGACCAAGCACCTACTGTTCCTGCACCTCAAGAATTCAATACTGCTATTACCAAAAGAAGTATTATTGGAATTATTATGGGTATTCTTGGCAACATTCCACAAGTAATCCAAATCATCATGAGTATTGTCAAAGCTTTCAAAGGTAACGTGAGTGGGTGGCGTTTGTTTAAGAAGATTAGCAAGAGAGAAGATATTGATTCTGTTGTTGCTGGTATCATTGCTGATATGCCATTTGTTGTCAGAGCTGTTGACACAGCCATGACTTCTGTTGCTTCTACCAAGAGAGATGGAGCTAATGATGACGTTGCTAATGCCGTCGTCAGATTGCCAGAAATTGTTGCTCGTGTTGCCACTGGTGTTCAACAATCCATCGAAAATGCCAAGAGAGATGGCGTTCCAGATGTTGGCCTTAATCTTGTTGCTAATGCTCCAAGACTTATCTCTAACGTTTTTGATGGCGTCCTGGAAACTGTTCAACAAGCTAAGAGAGATGGTCTTGAAGATTTTCTTGATGAACTTCTTCAAAGACTCCCACAACTCATTACTAGATCAGCTGAATCTGCTTTGAAAGACAGTCAACCAGTTAAAAGAGATGCCGGCTCAGTAGCACTTAGCAATTTAATCAAAAAGAGCATTGAAACTGTCGGTATTGAAAATGCTGCTCAAATTGTTTCAGAAAGAGATATTTCTTCTTTGATTGAAGAATATTTCGGAAAAGCTTAAACGCGTTCAGGTGCTGCCATGTTCTTTGCT |
| WOVP2HBT | CGCAGTTACGGATCAGTCACTCAGTCGACATGAAATTCTCCAAAATTGCCTGTGCTACTGTTTTTGCTTTATCTTCTCAAGCTGCCATCATCCACCATGCTCCAGAATTCAACATGAAGAGAGATGTTGTCCCAGCTGGTCAAGGTGATCCAGCCTCTGGCCCAGAACCTCAACTTGCTCCTGCACCTCAAGGAATCAACACTGATCTTGCCAAAAGAAGTATTATTGGAATTATTATGGGTATTCTTGGCAACATTCCACAAGTAATCCAAATCATCATGAGTATTGTCAAAGCTTTCAAAGGTAACGTGAGTGGGTGGCGTTTGTTTAAGAAGATTAGCAAGAGAGAAGATATTGATTCTGTTGTTGCTGGTATCATTGCTGATATGCCATTTGTTGTCAGAGCTGTTGACACAGCCATGACTTCTGTTGCTTCTACCAAGAGAGATGGAGCTAATGATGACGTTGCTAATGCCGTCGTCAGATTGCCAGAAATTGTTGCTCGTGTTGCCACTGGTGTTCAACAATCCATCGAAAATGCCAAGAGAGATGGCGTTCCAGATGTTGGCCTTAATCTTGTTGCTAATGCTCCAAGACTTATCTCTAACGTTTTTGATGGCGTCCTGGAAACTGTTCAACAAGCTAAGAGAGATGGTCTTGAAGATTTTCTTGATGAACTTCTTCAAAGACTCCCACAACTCATTACTAGATCAGCTGAATCTGCTTTGAAAGACAGTCAACCAGTTAAAAGAGATGCCGGCTCAGTAGCACTTAGCAATTTAATCAAAAAGAGCATTGAAACTGTCGGTATTGAAAATGCTGCTCAAATTGTTTCAGAAAGAGATATTTCTTCTTTGATTGAAGAATATTTCGGAAAAGCTTAAACGCGTTCAGGTGCTGCCATGTTCTTTGCT |
| WOVP3HBT | CGCAGTTACGGATCAGTCACTCAGTCGACATGAAATTCTCCAAAATTGCCTGTGCTACTGTTTTTGCTTTATCTTCTCAAGCTGCCATCATCCACCATGCTCCAGAATTCAACATGAAGAGAGATGTTGCTCCAGCTGCCCCAGCTGCTCCAGCTGACCAAGCACCTACTGTTCCTGCACCTCAAGAATTCAATACTGCTATTACCAAAAGAAGTTTTCTTAGTATTATCACTGCTCTTCTTGGAAACATTCCACAAATAATCCAAATCATCATGGGCATTGTCAAAGCTTTCAGAGGTAACGTGAGTGGGTGGCGTTTGTTTAAGAAGATTAGCAAGAGAGAAGATATTGATTCTGTTGTTGCTGGTATCATTGCTGATATGCCATTTGTTGTCAGAGCTGTTGACACAGCCATGACTTCTGTTGCTTCTACCAAGAGAGATGGAGCTAATGATGACGTTGCTAATGCCGTCGTCAGATTGCCAGAAATTGTTGCTCGTGTTGCCACTGGTGTTCAACAATCCATCGAAAATGCCAAGAGAGATGGCGTTCCAGATGTTGGCCTTAATCTTGTTGCTAATGCTCCAAGACTTATCTCTAACGTTTTTGATGGCGTCCTGGAAACTGTTCAACAAGCTAAGAGAGATGGTCTTGAAGATTTTCTTGATGAACTTCTTCAAAGACTCCCACAACTCATTACTAGATCAGCTGAATCTGCTTTGAAAGACAGTCAACCAGTTAAAAGAGATGCCGGCTCAGTAGCACTTAGCAATTTAATCAAAAAGAGCATTGAAACTGTCGGTATTGAAAATGCTGCTCAAATTGTTTCAGAAAGAGATATTTCTTCTTTGATTGAAGAATATTTCGGAAAAGCTTAAACGCGTTCAGGTGCTGCCATGTTCTTTGCT |
| WOVP2P3HBT | CGCAGTTACGGATCAGTCACTCAGTCGACATGAAATTCTCCAAAATTGCCTGTGCTACTGTTTTTGCTTTATCTTCTCAAGCTGCCATCATCCACCATGCTCCAGAATTCAACATGAAGAGAGATGTTGTCCCAGCTGGTCAAGGTGATCCAGCCTCTGGCCCAGAACCTCAACTTGCTCCTGCACCTCAAGGAATCAACACTGATCTTGCCAAAAGAAGTTTTCTTAGTATTATCACTGCTCTTCTTGGAAACATTCCACAAATAATCCAAATCATCATGGGCATTGTCAAAGCTTTCAGAGGTAACGTGAGTGGGTGGCGTTTGTTTAAGAAGATTAGCAAGAGAGAAGATATTGATTCTGTTGTTGCTGGTATCATTGCTGATATGCCATTTGTTGTCAGAGCTGTTGACACAGCCATGACTTCTGTTGCTTCTACCAAGAGAGATGGAGCTAATGATGACGTTGCTAATGCCGTCGTCAGATTGCCAGAAATTGTTGCTCGTGTTGCCACTGGTGTTCAACAATCCATCGAAAATGCCAAGAGAGATGGCGTTCCAGATGTTGGCCTTAATCTTGTTGCTAATGCTCCAAGACTTATCTCTAACGTTTTTGATGGCGTCCTGGAAACTGTTCAACAAGCTAAGAGAGATGGTCTTGAAGATTTTCTTGATGAACTTCTTCAAAGACTCCCACAACTCATTACTAGATCAGCTGAATCTGCTTTGAAAGACAGTCAACCAGTTAAAAGAGATGCCGGCTCAGTAGCACTTAGCAATTTAATCAAAAAGAGCATTGAAACTGTCGGTATTGAAAATGCTGCTCAAATTGTTTCAGAAAGAGATATTTCTTCTTTGATTGAAGAATATTTCGGAAAAGCTTAAACGCGTTCAGGTGCTGCCATGTTCTTTGCT |
| VOHBT | TCGCAGTTACGGATCAGTCACTCAGTCGACATGAAATTCTCCAAAATTGCCTGTGCTACTGTTTTTGCTTTATCTTCTCAAGCTGCCATCATTCACCATGCTCCAGAATTCAACATGAAGAGAGATGTTGTCCCAGCTGGTCAAGGTGATCCAGCCTCTGGCCCAGAACCTCAACTTGCTCCTGCACCTCAAGGAATCAACACTGATCTTGCCAAAAGAAGTTTTCTTAGTATTATCACTGCTCTTCTTGGAAACATTCCACAAATAATCCAAATCATCATGGGCATTGTCAAAGCTTTCAGAGGTAACGTGAGTGGGTGGCGTTTGTTTAAGAAGATTAGCAAGAGAGAAGATATTGATTCTGTTGTTGCTGGTATCATTGCTGATATGCCATTTGTTGCTAGAGCTGTTGACACAGCCATGACTTCTGTTGCTTCCACCAAGAGAGATGGAGCTAACGATGACGTTGCTAATGCCGTCGTCAGATTGCCAGAAATTGTTGCTCGTGTTGCCACTGGTGTTCAACAATCCATCGAAAATGCCAAGAGAGATGGCGTTCCAGACGTTGGTCTTAATCTTGTTGCTAATGCCCCAAGACTTATCTCTGACGTTTTTGATGGCGTCCTGGAAACTGTTCAACAAGCTAAGAGAGATGGTCTTGAAGATGCTCTTAATGAACTTCTTGAGCAACTCCCAAAACTTATTACTAGATCGGCTGAATCTGCTTTGAAAGACAGTCAACCAGTTAAAAGAGATGCCGGTTCAGTAGCACTTAGCAATTTAATTAAGAAAAGTATTGAAACTGTCGGTATTGAAAATGCTGCTCAAATTGTTTCAGAAAGAGATATTTCTTCTTTGATTGAAGAATATTTCGGAAATGCTTAAACGCGTTCAGGTGCTGCCATGTTCTTTGCT |
| VOWP2HBT | CGCAGTTACGGATCAGTCACTCAGTCGACATGAAATTCTCCAAAATTGCCTGTGCTACTGTTTTTGCTTTATCTTCTCAAGCTGCCATCATTCACCATGCTCCAGAATTCAACATGAAGAGAGATGTTGCTCCAGCTGCCCCAGCTGCTCCAGCTGACCAAGCACCTACTGTTCCTGCACCTCAAGAATTCAATACTGCTATTACCAAAAGAAGTTTTCTTAGTATTATCACTGCTCTTCTTGGAAACATTCCACAAATAATCCAAATCATCATGGGCATTGTCAAAGCTTTCAGAGGTAACGTGAGTGGGTGGCGTTTGTTTAAGAAGATTAGCAAGAGAGAAGATATTGATTCTGTTGTTGCTGGTATCATTGCTGATATGCCATTTGTTGCTAGAGCTGTTGACACAGCCATGACTTCTGTTGCTTCCACCAAGAGAGATGGAGCTAACGATGACGTTGCTAATGCCGTCGTCAGATTGCCAGAAATTGTTGCTCGTGTTGCCACTGGTGTTCAACAATCCATCGAAAATGCCAAGAGAGATGGCGTTCCAGACGTTGGTCTTAATCTTGTTGCTAATGCCCCAAGACTTATCTCTGACGTTTTTGATGGCGTCCTGGAAACTGTTCAACAAGCTAAGAGAGATGGTCTTGAAGATGCTCTTAATGAACTTCTTGAGCAACTCCCAAAACTTATTACTAGATCGGCTGAATCTGCTTTGAAAGACAGTCAACCAGTTAAAAGAGATGCCGGTTCAGTAGCACTTAGCAATTTAATTAAGAAAAGTATTGAAACTGTCGGTATTGAAAATGCTGCTCAAATTGTTTCAGAAAGAGATATTTCTTCTTTGATTGAAGAATATTTCGGAAATGCTTAAACGCGTTCAGGTGCTGCCATGTTCTTTGCT |
| VOWP3HBT | CGCAGTTACGGATCAGTCACTCAGTCGACATGAAATTCTCCAAAATTGCCTGTGCTACTGTTTTTGCTTTATCTTCTCAAGCTGCCATCATTCACCATGCTCCAGAATTCAACATGAAGAGAGATGTTGTCCCAGCTGGTCAAGGTGATCCAGCCTCTGGCCCAGAACCTCAACTTGCTCCTGCACCTCAAGGAATCAACACTGATCTTGCCAAAAGAAGTATTATTGGAATTATTATGGGTATTCTTGGCAACATTCCACAAGTAATCCAAATCATCATGAGTATTGTCAAAGCTTTCAAAGGTAACGTGAGTGGGTGGCGTTTGTTTAAGAAGATTAGCAAGAGAGAAGATATTGATTCTGTTGTTGCTGGTATCATTGCTGATATGCCATTTGTTGCTAGAGCTGTTGACACAGCCATGACTTCTGTTGCTTCCACCAAGAGAGATGGAGCTAACGATGACGTTGCTAATGCCGTCGTCAGATTGCCAGAAATTGTTGCTCGTGTTGCCACTGGTGTTCAACAATCCATCGAAAATGCCAAGAGAGATGGCGTTCCAGACGTTGGTCTTAATCTTGTTGCTAATGCCCCAAGACTTATCTCTGACGTTTTTGATGGCGTCCTGGAAACTGTTCAACAAGCTAAGAGAGATGGTCTTGAAGATGCTCTTAATGAACTTCTTGAGCAACTCCCAAAACTTATTACTAGATCGGCTGAATCTGCTTTGAAAGACAGTCAACCAGTTAAAAGAGATGCCGGTTCAGTAGCACTTAGCAATTTAATTAAGAAAAGTATTGAAACTGTCGGTATTGAAAATGCTGCTCAAATTGTTTCAGAAAGAGATATTTCTTCTTTGATTGAAGAATATTTCGGAAATGCTTAAACGCGTTCAGGTGCTGCCATGTTCTTTGCT |
| VOWP2P3HBT | TCGCAGTTACGGATCAGTCACTCAGTCGACATGAAATTCTCCAAAATTGCCTGTGCTACTGTTTTTGCTTTATCTTCTCAAGCTGCCATCATTCACCATGCTCCAGAATTCAACATGAAGAGAGATGTTGCTCCAGCTGCCCCAGCTGCTCCAGCTGACCAAGCACCTACTGTTCCTGCACCTCAAGAATTCAATACTGCTATTACCAAAAGAAGTATTATTGGAATTATTATGGGTATTCTTGGCAACATTCCACAAGTAATCCAAATCATCATGAGTATTGTCAAAGCTTTCAAAGGTAACGTGAGTGGGTGGCGTTTGTTTAAGAAGATTAGCAAGAGAGAAGATATTGATTCTGTTGTTGCTGGTATCATTGCTGATATGCCATTTGTTGCTAGAGCTGTTGACACAGCCATGACTTCTGTTGCTTCCACCAAGAGAGATGGAGCTAACGATGACGTTGCTAATGCCGTCGTCAGATTGCCAGAAATTGTTGCTCGTGTTGCCACTGGTGTTCAACAATCCATCGAAAATGCCAAGAGAGATGGCGTTCCAGACGTTGGTCTTAATCTTGTTGCTAATGCCCCAAGACTTATCTCTGACGTTTTTGATGGCGTCCTGGAAACTGTTCAACAAGCTAAGAGAGATGGTCTTGAAGATGCTCTTAATGAACTTCTTGAGCAACTCCCAAAACTTATTACTAGATCGGCTGAATCTGCTTTGAAAGACAGTCAACCAGTTAAAAGAGATGCCGGTTCAGTAGCACTTAGCAATTTAATTAAGAAAAGTATTGAAACTGTCGGTATTGAAAATGCTGCTCAAATTGTTTCAGAAAGAGATATTTCTTCTTTGATTGAAGAATATTTCGGAAATGCTTAAACGCGTTCAGGTGCTGCCATGTTCTTTGCT |
