# Supplementary material for: A variant ECE1 allele contributes to reduced pathogenicity of Candida albicans during vulvovaginal candidiasis
Source: PLoS Pathog. 2021 Sep 10;17(9):e1009884. doi: 10.1371/journal.ppat.1009884 (PMC8432879; doi:10.1371/journal.ppat.1009884)
Supplement: S4 Table — (DOCX) [file ppat.1009884.s007.docx]

**S4 Table. *ECE1* P2 and candidalysin sequences from *C. albicans* clinical isolates.** Sanger sequencing of global clinical isolates (n=78) were aligned using Clustal Omega and grouped as SC5314- and 529L-like. KR repeats are bolded.

| **Strain no.** | **Source** | **ECE1 P2-P3 Sequencing** |
| --- | --- | --- |
| SC5314 | Blood culture | **KR**DVAPAAPAAPADQAPTVPAPQEFNTAIT**KR**SIIGIIMGILGNIPQVIQIIMSIVKAFKGN**KR** |
| FAHJU-2020-932 | Reproductive tract | **KR**DVAPAAPAAPADQAPTVPAPQEFNTAIT**KR**SIIGIIMGILGNIPQVIQIIMSIVKAFKGN**KR** |
| FAHGMU-2019-047 | Sterile body fluid | **KR**DVAPAAPAAPADQAPTVPAPQEFNTAIT**KR**SIIGIIMGILGNIPQVIQIIMSIVKAFKGN**KR** |
| TAHSYU-2020-123 | Reproductive tract | **KR**DVAPAAPAAPADQAPTVPAPQEFNTAIT**KR**SIIGIIMGILGNIPQVIQIIMSIVKAFKGN**KR** |
| ZHSMU-2019-242 | Wounds | **KR**DVAPAAPAAPADQAPTVPAPQEFNTAIT**KR**SIIGIIMGILGNIPQVIQIIMSIVKAFKGN**KR** |
| ZHSMU-2019-848 | Blood culture | **KR**DVAPAAPAAPADQAPTVPAPQEFNTAIT**KR**SIIGIIMGILGNIPQVIQIIMSIVKAFKGN**KR** |
| GDHTCM-2019-079 | Reproductive tract | **KR**DVAPAAPAAPADQAPTVPAPQEFNTAIT**KR**SIIGIIMGILGNIPQVIQIIMSIVKAFKGN**KR** |
| TAHSYU-2020-006 | Reproductive tract | **KR**DVAPAAPAAPADQAPTVPAPQEFNTAIT**KR**SIIGIIMGILGNIPQVIQIIMSIVKAFKGN**KR** |
| FAHJU-2020-432 | Reproductive tract | **KR**DVAPAAPAAPADQAPTVPAPQEFNTAIT**KR**SIIGIIMGILGNIPQVIQIIMSIVKAFKGN**KR** |
| GDHTCM-2019-084 | Reproductive tract | **KR**DVAPAAPAAPADQAPTVPAPQEFNTAIT**KR**SIIGIIMGILGNIPQVIQIIMSIVKAFKGN**KR** |
| FAHJU-2020-220 | Reproductive tract | **KR**DVAPAAPAAPADQAPTVPAPQEFNTAIT**KR**SIIGIIMGILGNIPQVIQIIMSIVKAFKGN**KR** |
| FAHGMU-2019-049 | Sterile body fluid | **KR**DVAPAAPAAPADQAPTVPAPQEFNTAIT**KR**SIIGIIMGILGNIPQVIQIIMSIVKAFKGN**KR** |
| GDHTCM-2019-096 | Reproductive tract | **KR**DVAPAAPAAPADQAPTVPAPQEFNTAIT**KR**SIIGIIMGILGNIPQVIQIIMSIVKAFKGN**KR** |
| FAHJU-2020-546 | Reproductive tract | **KR**DVAPAAPAAPADQAPTVPAPQEFNTAIT**KR**SIIGIIMGILGNIPQVIQIIMSIVKAFKGN**KR** |
| ZHSMU-2019-356 | Others | **KR**DVAPAAPAAPADQAPTVPAPQEFNTAIT**KR**SIIGIIMGILGNIPQVIQIIMSIVKAFKGN**KR** |
| FAHJU-2020-314 | Reproductive tract | **KR**DVAPAAPAAPADQAPTVPAPQEFNTAIT**KR**SIIGIIMGILGNIPQVIQIIMSIVKAFKGN**KR** |
| FAHJU-2020-027 | Reproductive tract | **KR**DVAPAAPAAPADQAPTVPAPQEFNTAIT**KR**SIIGIIMGILGNIPQVIQIIMSIVKAFKGN**KR** |
| GDHTCM-2020-032 | Reproductive tract | **KR**DVAPAAPAAPADQAPTVPAPQEFNTAIT**KR**SIIGIIMGILGNIPQVIQIIMSIVKAFKGN**KR** |
| FAHJU-2020-556 | Reproductive tract | **KR**DVAPAAPAAPADQAPTVPAPQEFNTAIT**KR**SIIGIIMGILGNIPQVIQIIMSIVKAFKGN**KR** |
| FAHGMU-2019-086 | Sterile body fluid | **KR**DVAPAAPAAPADQAPTVPAPQEFNTAIT**KR**SIIGIIMGILGNIPQVIQIIMSIVKAFKGN**KR** |
| ZHSMU-2019-135 | Wounds | **KR**DVAPAAPAAPADQAPTVPAPQEFNTAIT**KR**SIIGIIMGILGNIPQVIQIIMSIVKAFKGN**KR** |
| JS21 | Reproductive | **KR**DVAPAAPAAPADQAPTVPAPQEFNTAIT**KR**SIIGIIMGILGNIPQVIQIIMSIVKAFKGN**KR** |
| JS24 | Reproductive | **KR**DVAPAAPAAPADQAPTVPAPQEFNTAIT**KR**SIIGIIMGILGNIPQVIQIIMSIVKAFKGN**KR** |
| JS19 | Reproductive | **KR**DVAPAAPAAPADQAPTVPAPQEFNTAIT**KR**SIIGIIMGILGNIPQVIQIIMSIVKAFKGN**KR** |
| JS16 | Reproductive | **KR**DVAPAAPAAPADQAPTVPAPQEFNTAIT**KR**SIIGIIMGILGNIPQVIQIIMSIVKAFKGN**KR** |
| JS13 | Reproductive | **KR**DVAPAAPAAPADQAPTVPAPQEFNTAIT**KR**SIIGIIMGILGNIPQVIQIIMSIVKAFKGNKR |
| JS14 | Reproductive | **KR**DVAPAAPAAPADQAPTVPAPQEFNTAIT**KR**SIIGIIMGILGNIPQVIQIIMSIVKAFKGN**KR** |
| FAHJU-2020-037 | Reproductive tract | **KR**DVAPAAPAAPADQAPTVPAPQEFNTDIT**KR**SIIGIIMGILGNIPQVIQIIMSIVKAFKGN**KR** |
| TAHSYU-2020-128 | Reproductive tract | **KR**DVAPAAPAAPADQAPTVPAPQEFNTDIT**KR**SIIGIIMGILGNIPQVIQIIMSIVKAFKGN**KR** |
| FAHGMU-2019-072 | Sterile body fluid | **KR**DVAPAAPAAPADQAPTVPAPQEFNTDIT**KR**SIIGIIMGILGNIPQVIQIIMSIVKAFKGN**KR** |
| FAHGMU-2019-073 | Sterile body fluid | **KR**DVAPAAPAAPADQAPTVPAPQEFNTDIT**KR**SIIRIIMGILGNIPQVIQIIMSIVKAFKGN**KR** |
| FAHJU-2020-452 | Reproductive tract | **KR**DVAPAAPAAPADQAPTVPAPQEFNTDIT**KR**SIIRIIMGILGNIPQVIQIIMSIVKAFKGN**KR** |
| FAHJU-2020-511 | Reproductive tract | **KR**DVAPDAPAAPGEQAHTVPAPQEFNTDIT**KR**SIIGIIMGILGNIPQVIQIIMSIVKAFKGN**KR** |
| TAHSYU-2020-016 | Reproductive tract | **KR**DVAPDAPAAPGEQAHTVPAPQEFNTDIT**KR**SIIGIIMGILGNIPQVIQIIMSIVKAFKGN**KR** |
| 529L | Oral cavity | **KR**DVVPAGQGDPASGPEPQLAPAPQGINTDLA**KR**SFLSIITALLGNIPQIIQIIMGIVKAFRGN**KR** |
| ZHSMU-2019-334 | Sterile body fluid | **KR**DVVPAGQGDPASGPEPQLAPAPQGINTDLA**KR**SFLSIITALLGNIPQIIQIIMGIVKAFRGN**KR** |
| GDHTCM-2019-052 | Reproductive tract | **KR**DVVPAGQGDPASGPEPQLAPAPQGINTDLA**KR**SFLSIITALLGNIPQIIQIIMGIVKAFRGN**KR** |
| FAHGMU-2018-086 | Sterile body fluid | **KR**DVVPAGQGDPASGPEPQLAPAPQGINTDLA**KR**SFLSIITALLGNIPQIIQIIMGIVKAFRGN**KR** |
| FAHGMU-2018-088 | Sterile body fluid | **KR**DVVPAGQGDPASGPEPQLAPAPQGINTDLA**KR**SFLSIITALLGNIPQIIQIIMGIVKAFRGN**KR** |
| ZHSMU-2019-134 | Sterile body fluid | **KR**DVVPAGQGDPASGPEPQLAPAPQGINTDLA**KR**SFLSIITALLGNIPQIIQIIMGIVKAFRGN**KR** |
| GDHTCM-2020-002 | Reproductive tract | **KR**DVVPAGQGDPASGPEPQLAPAPQGINTDLA**KR**SFLSIITALLGNIPQIIQIIMGIVKAFRGN**KR** |
| TAHSYU-2020-069 | Reproductive tract | **KR**DVVPAGQGDPASGPEPQLAPAPQGINTDLA**KR**SFLSIITALLGNIPQIIQIIMGIVKAFRGN**KR** |
| FAHGMU-2018-075 | Blood culture | **KR**DVVPAGQGDPASGPEPQLAPAPQGINTDLA**KR**SFLSIITALLGNIPQIIQIIMGIVKAFRGN**KR** |
| GDHTCM-2020-014 | Reproductive tract | **KR**DVVPAGQGDPASGPEPQLAPAPQGINTDLA**KR**SFLSIITALLGNIPQIIQIIMGIVKAFRGN**KR** |
| FAHGMU-2019-135 | Sterile body fluid | **KR**DVVPAGQGDPASGPEPQLAPAPQGINTDLA**KR**SFLSIITALLGNIPQIIQIIMGIVKAFRGN**KR** |
| ZHSMU-2019-281 | Sterile body fluid | **KR**DVVPAGQGDPASGPEPQLAPAPQGINTDLA**KR**SFLSIITALLGNIPQIIQIIMGIVKAFRGN**KR** |
| TAHSYU-2020-086 | Reproductive tract | **KR**DVVPAGQGDPASGPEPQLAPAPQGINTDLA**KR**SFLSIITALLGNIPQIIQIIMGIVKAFRGN**KR** |
| GDHTCM-2020-053 | Reproductive tract | **KR**DVVPAGQGDPASGPEPQLAPAPQGINTDLA**KR**SFLSIITALLGNIPQIIQIIMGIVKAFRGN**KR** |
| ZHSMU-2019-612 | Sterile body fluid | **KR**DVVPAGQGDPASGPEPQLAPAPQGINTDLA**KR**SFLSIITALLGNIPQIIQIIMGIVKAFRGN**KR** |
| ZHSMU-2019-566 | Blood culture | **KR**DVVPAGQGDPASGPEPQLAPAPQGINTDLA**KR**SFLSIITALLGNIPQIIQIIMGIVKAFRGN**KR** |
| FAHGMU-2019-023 | Sterile body fluid | **KR**DVVPAGQGDPASGPEPQLAPAPQGINTDLA**KR**SFLSIITALLGNIPQIIQIIMGIVKAFRGN**KR** |
| FAHJU-2020-473 | Reproductive tract | **KR**DVVPAGQGDPASGPEPQLAPAPQGINTDLA**KR**SFLSIITALLGNIPQIIQIIMGIVKAFRGN**KR** |
| TAHSYU-2020-137 | Reproductive tract | **KR**DVVPAGQGDPASGPEPQLAPAPQGINTDLA**KR**SFLSIITALLGNIPQIIQIIMGIVKAFRGN**KR** |
| ZHSMU-2019-294 | Blood culture | **KR**DVVPAGQGDPASGPEPQLAPAPQGINTDLA**KR**SFLSIITALLGNIPQIIQIIMGIVKAFRGN**KR** |
| GDHTCM-2020-060 | Reproductive tract | **KR**DVVPAGQGDPASGPEPQLAPAPQGINTDLA**KR**SFLSIITALLGNIPQIIQIIMGIVKAFRGN**KR** |
| FAHJU-2020-555 | Reproductive tract | **KR**DVVPAGQGDPASGPEPQLAPAPQGINTDLA**KR**SFLSIITALLGNIPQIIQIIMGIVKAFRGN**KR** |
| TAHSYU-2020-051 | Reproductive tract | **KR**DVVPAGQGDPASGPEPQLAPAPQGINTDLA**KR**SFLSIITALLGNIPQIIQIIMGIVKAFRGN**KR** |
| TAHSYU-2020-052 | Reproductive tract | **KR**DVVPAGQGDPASGPEPQLAPAPQGINTDLA**KR**SFLSIITALLGNIPQIIQIIMGIVKAFRGN**KR** |
| GDHTCM-2019-070 | Reproductive tract | **KR**DVVPAGQGDPASGPEPQLAPAPQGINTDLA**KR**SFLSIITALLGNIPQIIQIIMGIVKAFRGN**KR** |
| FAHJU-2020-697 | Reproductive tract | **KR**DVVPAGQGDPASGPEPQLAPAPQGINTDLA**KR**SFLSIITALLGNIPQIIQIIMGIVKAFRGN**KR** |
| FAHJU-2020-537 | Reproductive tract | **KR**DVVPAGQGDPASGPEPQLAPAPQGINTDLA**KR**SFLSIITALLGNIPQIIQIIMGIVKAFRGN**KR** |
| ZHSMU-2019-753 | Sterile body fluid | **KR**DVVPAGQGDPASGPEPQLAPAPQGINTDLA**KR**SFLSIITALLGNIPQIIQIIMGIVKAFRGN**KR** |
| TAHSYU-2020-102 | Reproductive tract | **KR**DVVPAGQGDPASGPEPQLAPAPQGINTDLA**KR**SFLSIITALLGNIPQIIQIIMGIVKAFRGN**KR** |
| TAHSYU-2020-104 | Reproductive tract | **KR**DVVPAGQGDPASGPEPQLAPAPQGINTDLA**KR**SFLSIITALLGNIPQIIQIIMGIVKAFRGN**KR** |
| GDHTCM-2020-038 | Reproductive tract | **KR**DVVPAGQGDPASGPEPQLAPAPQGINTDLA**KR**SFLSIITALLGNIPQIIQIIMGIVKAFRGN**KR** |
| ZHSMU-2019-166 | Others | **KR**DVVPAGQGDPASGPEPQLAPAPQGINTDLA**KR**SFLSIITALLGNIPQIIQIIMGIVKAFRGN**KR** |
| FAHJU-2020-015 | Reproductive tract | **KR**DVVPAGQGDPASGPEPQLAPAPQGINTDLA**KR**SFLSIITALLGNIPQIIQIIMGIVKAFRGN**KR** |
| GDHTCM-2020-041 | Reproductive tract | **KR**DVVPAGQGDPASGPEPQLAPAPQGINTDLA**KR**SFLSIITALLGNIPQIIQIIMGIVKAFRGN**KR** |
| TAHSYU-2020-111 | Reproductive tract | **KR**DVVPAGQGDPASGPEPQLAPAPQGINTDLA**KR**SFLSIITALLGNIPQIIQIIMGIVKAFRGN**KR** |
| FAHGMU-2019-042 | Sterile body fluid | **KR**DVVPAGQGDPASGPEPQLAPAPQGINTDLA**KR**SFLSIITALLGNIPQIIQIIMGIVKAFRGN**KR** |
| JS06 | Reproductive | **KR**DVVPAGQGDPASGPEPQLAPAPQGINTDLA**KR**SFLSIITALLGNIPQIIQIIMGIVKAFRGN**KR** |
| JS10 | Reproductive | **KR**DVVPAGQGDPASGPEPQLAPAPQGINTDLA**KR**SFLSIITALLGNIPQIIQIIMGIVKAFRGN**KR** |
| JS12 | Reproductive | **KR**DVVPAGQGDPASGPEPQLAPAPQGINTDLA**KR**SFLSIITALLGNIPQIIQIIMGIVKAFRGN**KR** |
| JS22 | Reproductive | **KR**DVVPAGQGDPASGPEPQLAPAPQGINTDLA**KR**SFLSIITALLGNIPQIIQIIMGIVKAFRGN**KR** |
| JS23 | Reproductive | **KR**DVVPAGQGDPASGPEPQLAPAPQGINTDLA**KR**SFLSIITALLGNIPQIIQIIMGIVKAFRGN**KR** |
| JS27 | Reproductive | **KR**DVVPAGQGDPASGPEPQLAPAPQGINTDLA**KR**SFLSIITALLGNIPQIIQIIMGIVKAFRGN**KR** |
| FAHJU-2019-410 | Sterile body fluid | **KR**DVVPAGQGDPASGPEPQLAPAPQGINTDLA**KR**SFLSIITALLGNIPQIIQIIMGIVKAFRGN**KR** |
| FAHJU-2019-311 | Sterile body fluid | **KR**DVVPAGQGDPASGPEPQLAPAPQGINTDLA**KR**SFLSIITALLGNIPQIIQIIMGIVKAFRGN**KR** |
| FAHJU-2019-934 | Respiratory tract | **KR**DVVPAGQGDPASGPEPQLAPAPQGINTDLA**KR**SFLSIITALLGNIPQIIQIIMGIVKAFRGN**KR** |
| FAHJU-2019-561 | Reproductive tract | **KR**DVVPAGQGDPASGPEPQLAPAPQGINTDLA**KR**SFLSIITALLGNIPQIIQIIMGIVKAFRGN**KR** |
